# Burden of tumor mutations, neoepitopes, and other variants are weak predictors of cancer immunotherapy response and overall survival

**DOI:** 10.1186/s13073-020-00729-2

**Published:** 2020-03-30

**Authors:** Mary A. Wood, Benjamin R. Weeder, Julianne K. David, Abhinav Nellore, Reid F. Thompson

**Affiliations:** 1 Computational Biology Program, Oregon Health & Science University, Portland, USA; 2 Portland VA Research Foundation, Portland, USA; 3 Department of Biomedical Engineering, Oregon Health & Science University, Portland, USA; 4 Department of Surgery, Oregon Health & Science University, Portland, USA; 5 Department of Radiation Medicine, Oregon Health & Science University, Portland, USA; 6 Department of Medical Informatics & Clinical Epidemiology, Oregon Health & Science University, Portland, USA; 7 VA Portland Healthcare System, Division of Hospital and Specialty Medicine, Portland, USA

**Keywords:** Tumor mutational burden, TMB, Neoepitopes, Neoepitope burden, Neoantigens, Splice junctions, Retained introns, Tumor variant burden, Immunotherapy response

## Abstract

**Background:**

Tumor mutational burden (TMB; the quantity of aberrant nucleotide sequences a given tumor may harbor) has been associated with response to immune checkpoint inhibitor therapy and is gaining broad acceptance as a result. However, TMB harbors intrinsic variability across cancer types, and its assessment and interpretation are poorly standardized.

**Methods:**

Using a standardized approach, we quantify the robustness of TMB as a metric and its potential as a predictor of immunotherapy response and survival among a diverse cohort of cancer patients. We also explore the additive predictive potential of RNA-derived variants and neoepitope burden, incorporating several novel metrics of immunogenic potential.

**Results:**

We find that TMB is a partial predictor of immunotherapy response in melanoma and non-small cell lung cancer, but not renal cell carcinoma. We find that TMB is predictive of overall survival in melanoma patients receiving immunotherapy, but not in an immunotherapy-naive population. We also find that it is an unstable metric with potentially problematic repercussions for clinical cohort classification. We finally note minimal additional predictive benefit to assessing neoepitope burden or its bulk derivatives, including RNA-derived sources of neoepitopes.

**Conclusions:**

We find sufficient cause to suggest that the predictive clinical value of TMB should not be overstated or oversimplified. While it is readily quantified, TMB is at best a limited surrogate biomarker of immunotherapy response. The data do not support isolated use of TMB in renal cell carcinoma.

**Electronic supplementary material:**

The online version of this article (10.1186/s13073-020-00729-2) contains supplementary material, which is available to authorized users.

## Background

The advent of immunotherapy as a promising form of cancer treatment has been accompanied by a parallel effort to explore potential mechanisms and drivers of therapeutic response. For instance, tumor mutational burden (TMB; the overall quantity of aberrant nucleotide sequences a given tumor may harbor) has been associated with response to immune checkpoint inhibitor therapy [1] and overall survival [2]. Similarly, the quantity of nonsynonymous single nucleotide variants was shown to be associated with immunotherapy response in several independent clinical cohorts [3–6]. Other sources of sequence variation such as frameshifting insertions/deletions [7] and tumor-specific alternative splicing (e.g., intron retention [8]) have also been found to correlate with immunotherapy response. These phenomena are widely accepted and appear to be particularly pronounced in patients harboring DNA repair deficiencies [9]. Indeed, the checkpoint inhibitor, pembrolizumab, was granted accelerated disease-agnostic approval by the FDA on this basis for any cancer patient-harboring deficiencies in their capacity to perform DNA mismatch repair [10]. Moreover, an expanding cohort of clinical immunotherapy trials (e.g., NCT03668119, NCT03178552, NCT03519412) are actively utilizing TMB status as a key inclusion criterion. However, there is wide variability among techniques for measuring and interpreting TMB, raising questions of utility and reproducibility [11].

Given the perceived critical importance of TMB in the research setting and its emerging role in the oncology clinic, we sought to quantify the robustness of TMB as a metric and explore its deeper nuances using pooled whole exome sequencing data from a variety of previously published studies. While TMB is generally correlated with downstream metrics such as neoepitope burden, we also explore the predictive capacity of neoepitope burden and its derivatives including adjustment for MHC binding robustness and peptide sequence novelty, as well as RNA-derived sources of neoepitopes.

## Methods

### Variant identification and neoepitope prediction

We assembled a cohort of 457 tumor samples from 431 different cancer patients from publicly available data, including 302 melanoma patients (326 tumor samples) [1, 4–6, 12–16], 34 non-small cell lung cancer (NSCLC) patients (34 tumor samples) [3], 10 prostate cancer patients (10 tumor samples) [17], 57 renal cell carcinoma (RCC) patients (58 tumor samples) [18], and 28 mismatch repair (MMR)-deficient (as determined by polymerase chain reaction or immunohistochemistry [9]) colon, endometrial, and thyroid cancer patients (29 tumor samples) [9] (see Additional file 1: Table S1). Despite attempts to obtain these data, we unfortunately were forced to omit tumor samples from 75 NSCLC patients [19], for whom data was not available due to limitations of patient consent at the time of the study. Alignment of whole exome sequencing (WES) reads was performed as described previously [20]. The Mbp of genome covered was determined using bedtools genomecov (v2.26.0) [21], where any base covered by a depth of at least six reads was considered covered, as this is twice the minimum read depth required for variant detection by SomaticSniper [22] and VarScan 2 [23]. Somatic and germline variant calling were performed as described previously [20]. To obtain coverage-adjusted mutation burdens for each patient, we divided the number of consensus somatic variants by the Mbp of genome covered by sequencing. We employed HapCUT2 for patient-specific haplotype phasing. To do this, germline and consensus somatic variants were combined into a single VCF using neoepiscope’s [20] (v0.3.5) merge functionality. HapCUT2’s extractHAIRS software was run with the merged VCF and the tumor alignment file, allowing for extraction of reads spanning indels, to produce the fragment file used with HapCUT2 to predict haplotypes. Neoepitopes of 8–24 amino acids in length were predicted for this cohort using neoepiscope, including background germline variation and variant phasing, and enumerating neoepitopes from protein coding, nonsense mediated decay, polymorphic pseudogene, T cell receptor variable, and immunoglobulin variable transcripts. Additionally, to better understand how the choice of variant caller impacts downstream neoepitope predictions, we ran neoepiscope excluding background germline variation and variant phasing separately for our consensus somatic variants and variants produced by individual variant calling tools, only enumerating neoepitopes from protein coding transcripts. For patients with multiple tumor samples, the median mutation and neoepitope burdens across samples were retained. Variants that were pathogenic or likely pathogenic in cancer according to ClinVar [24] were identified using Open-CRAVAT [25], and neoepitopes deriving from these variants were flagged. We used the software mSINGS [26] (bit bucket commit 030289381f3b7aee24d8eccbb69b3e66711f5bb0) to identify tumors with MSI-positive status. The software was run on each tumor alignment file, and the provided TCGA msi_bed, msi_baseline, msi_intervals were used.

### RNA variant identification

Among the overall cohort, 106 patients (89 melanoma patients [1, 4–6] and 17 RCC patients [18]) had complementary tumor RNA-sequencing (RNA-seq) data. We aligned RNA-seq reads to both the GRCh37d5 and GRCh38 genomes using STAR (v2.6.1c) [27], using the ‘intronMotif’ --outSAMstrandField option and specifying NH, HI, AS, nM, and MD fields with the --outSAMattributes option. To identify putative tumor-specific splice junctions, we first downloaded called junction data including coverage and bed files for TCGA and GTEx using recount2 [28]. GENCODE version 28 annotations [29] were downloaded and parsed to collect full coordinates and left and right splice sites of junctions from annotated transcripts. The TCGA phenotype file from Rail-RNA [30] was parsed to collect sample type (primary, recurrent, or metastatic tumor vs. matched normal). A new SQLite3 database was created to index all GTEx and TCGA junctions, with linked tables containing (1) sample ids and associated junction ids; (2) sample ids and phenotype information for each sample; and (3) junction ids and junction information including GENCODE annotation status and location within protein coding gene boundaries. Junctions were extracted from the SJ.out output files generated by STAR; only junctions with canonical splice motifs (GT-AG, GC-AG, and AT-AC) were collected. No minimum read count was imposed for a junction to be called in a sample. The known junction index was queried to collect all junctions found in normal tissue either in GTEx or in TCGA matched normal samples and these normal junctions were filtered out from the single-sample set. We used the MetaSRA [31] web query interface to collect Sequence Read Archive (SRA) accession numbers for non-cancerous melanocyte cell line [32] and primary cell [33] RNA-seq experiments. The resulting accession numbers were queried against the Snaptron junction database [34, 35] to download junctions from across the entire genome. All junctions found in these normal melanocyte samples as well as all fully GENCODE-annotated junctions were also eliminated from each single-sample junction set. Again, no minimum read support was required; a single read covering a junction in a single non-cancer sample (SRA, GTEx, or TCGA) eliminated the junction from the patient set. Finally, we removed junctions where neither end was found in GENCODE annotation, yielding a list of putative tumor-specific splice sites for each patient. Additional file 2: Figure S1 (rows 2–4) illustrates the variety of splicing alterations captured.

We identified tumor-specific retained introns (see Additional file 2: Figure S1, row 5) using Keep Me Around (kma) [36]. We aligned RNA-seq reads to a modified version of the GRCh37d5 using Bowtie 2 (v2.3.4.3) [37], and quantified reads using eXpress (v1.5.1) [38], as per kma recommendations. After computing intron retention, we used kma’s filters to retain only transcripts that were expressed at greater than or equal to 1 transcript per million (TPM) in at least 25% of samples, transcripts that had at least 5 unique counts in at least 25% of samples, and transcripts that had greater than 0 and less than 100% of introns retained. To prevent inclusion of artifacts from unprocessed transcripts, we identified outlier introns among the distribution of transcript read counts, only retaining introns with a read count greater than 3 median absolute deviations above the median intron read count for a transcript, and greater than or equal to the read count for the transcript itself. To filter out retained introns that may be expressed in normal tissues, we performed the same analysis using publically available RNA-seq reads from melanocyte samples of 106 newborns [39]. Any retained introns identified from the melanocyte RNA-seq data were then removed from the retained introns identified from the tumor RNA-seq data. Neoepitopes deriving from retained introns were predicted using the reading frame from the 5′ end of the transcript of origin prior to the intron, enumerating peptides 8–24 amino acids in length.

### HLA type prediction and related analyses

MHC Class I alleles for each patient were predicted from tumor WES reads using Optitype (v1.0) [40], and MHC Class II alleles for each patient were predicted from tumor WES reads using seq2hla (v2.2) [41]. For each neoepitope sequence predicted from phased variants (see above), a patient’s predicted MHC Class I and MHC Class II alleles were used for binding affinity predictions with MHCnuggets (v2.1) [42]. Neoepitopes were counted toward a patient’s neoepitope burden if they bound at least one of a patient’s MHC alleles with high affinity (≤ 500 nM). For comparison with neoepitope burdens reported by the authors of the five original manuscripts with reported neoepitope burdens, we tallied binding predictions separately based on their methodology, using binding affinity predictions from NetMHCpan (v4.0) [43] for a more direct comparison. For patients from the studies by Carreno et al. [4] and Rizvi et al. [3], we considered only 9mer epitopes; for patients from the study by Van Allen et al. [1], we considered only 9mer and 10mer epitopes; and for the studies by Hugo et al. [5] and Roh et al. [14], we considered 9mer, 10mer, and 11mer epitopes. For the epitopes from patients from the Carreno et al. study, we only considered binding to HLA-A*02:01 as in their paper, while for the other studies we considered binding to any MHC Class I epitope. Additionally, we determined the burden of processed neoepitopes (those predicted to be cleaved by the proteasome, transported by TAP, and presented on the cell surface by an MHC Class I molecule) using NetCTLpan (v1.1) [44]. For each tumor sample, we ran NetCTLpan predictions for all 8mer, 9mer, 10mer, and 11mer neopeptides with each MHC Class I epitope predicted by Optitype. A neopeptide was counted toward the burden of processed epitopes if its NetCTLpan combined score rank was in the top 1% for at least one MHC allele.

### Modified neoepitope burden

To better understand how different features of tumor neoepitopes might influence response to immunotherapy, we produced several normalized neoepitope burdens. We first calculated neoepitope burden for each patient weighted by MHC allele presentation, where a predicted neoepitope sequence counted toward the patient’s neoepitope burden once for each of the patient’s MHC alleles that was predicted to bind that neoepitope with high affinity (≤ 500 nM). Second, neoepitope burden was calculated for each patient weighted by amino acid mismatch as follows. The closest normal peptide in the human proteome to each neoepitope was identified using blastp (v2.6.0) [45], selecting for lowest E value or, in the case of a tie among multiple peptide sequences, the selected peptide was that with the highest weighted BLOSUM62 similarity (as described previously [46]). A neoepitope sequence was counted toward the patient’s neoepitope burden once for each amino acid mismatch between the neoepitope and its closest normal peptide. Third, neoepitope burden was calculated for each patient weighted by TCGA transcript expression of the transcript(s) of origin for each neoepitope. We identified expressed transcripts in matched TCGA cancer types for each disease type in our cohort (SKCM for melanoma, LUAD/LUSC for NSCLC, COAD for colon cancer, UCEC for endometrial cancer, THCA for thyroid cancer, PRAD for prostate cancer, and KIRC for RCC) from TPM values generated by the National Cancer Institute [47]. A transcript was considered “expressed” for a cancer type if the 75th quantile TPM value for that transcript in that disease was greater than 1 TPM. Because these TPM values were based on GRCh38 transcripts, we used liftOver [48] to convert the coordinates of a neoepitope’s mutation of origin to GRCh38 coordinates and identify overlapping transcripts. A neoepitope sequence was counted toward the patient’s neoepitope burden once for each transcript of origin expressed in TCGA. Note that for patients with tumor RNA-seq data (see above), we also calculated neoepitope burden weighted by patient-specific expression of the transcript(s) of origin for each neoepitope. We used Rail-RNA (v0.2.4b) [30] on RNA-seq alignments to the GRCh37d5 genome to identify covered exons, and a transcript was considered “expressed” if at least 1 read covered any exon in the transcript. A neoepitope sequence was counted toward the patient’s neoepitope burden once for each expressed transcript of origin. Finally, we multiplicatively combined these weighted burdens by multiplying scores for each epitope and totaling all epitope scores: allele presentation score by amino acid mismatch score, allele presentation score by TCGA expression score, allele presentation score by patient-specific expression score (if relevant), amino acid mismatch score by TCGA expression score, amino acid mismatch score by patient-specific expression score (if relevant), allele presentation score by amino acid mismatch score by TCGA expression score, and allele presentation score by amino acid mismatch score by patient-specific expression score (if relevant).

### Statistical analysis

Statistical analysis was performed in R (v3.5.1). The rlm function from the MASS package (v7.3-51.4) was used for robust linear model fitting, and the cor.test function was used for determining Pearson product-moment correlation values. To determine variability in TMB across variant calling tools, the median of pairwise differences in TMB between tools was divided by the median TMB across tools for each patient; the median of these values across patients was reported. The roc function from the pROC package (v1.14.0) was used to generate ROC curves for any predictors of immunotherapy response and to determine their AUC for all patients with reported immunotherapy response status (409/414, after excluding 3 colon cancer, 1 prostate cancer, and 1 RCC patient that lacked documented response status). Logistic regression was performed using the glm function to model therapeutic response as a linear function of TMB (on log scale), and neoepitopes (log scale) on the 245 melanoma patients, 50 RCC patients, and 33 NSCLC patients with reported immunotherapy response status to either aCTLA4 or aPD1 treatment alone (excluding dual/combination checkpoint inhibitor therapy). For the subset of these patients with available RNA-seq data (see Additional file 1: Table S1), tumor variant burden (TVB; the sum of somatic variants, tumor-specific splice junctions, and tumor-specific retained introns; log2 scale) was also modeled. The fit models were subsequently used to estimate the odds of therapeutic response at the 25th and 75th TMB, TVB, and neoepitope percentiles. Each cancer type was modeled separately, with the melanoma model accounting for differences in aCTLA4 vs. aPD1 response rates. *P* values were adjusted for multiple comparisons using the Benjamini-Hochberg method with the p.adjust function.

### Survival analysis

Due to the low number of observed events for some cancers, only melanoma and RCC patient cohorts were appropriate for survival analysis. Patients were included in survival analysis if they had information on both overall survival status, as well as either time to event or time to censorship data. In total, 218 melanoma patients and 56 RCC patients were selected for analysis in R (v3.5.1). The coxph function from the survival package (v2.44-1.1) was used to fit proportional hazards regression models, and the survfit function from the survival package was used to compute survival curves. For comparison with patients not treated with immunotherapy, we also performed survival analysis with SKCM and KIRC patients from TCGA. We obtained mutation annotation format (MAF) files and clinical data for these patients from the Broad Institute [49]. Patients with both mutation information and survival information were used for analysis (320 SKCM patients and 415 KIRC patients). Mutational burden was determined by counting the number of somatic variants listed in each patient’s MAF file, and a patient was considered to have survival information if they had information on time to death or a non-zero and non-negative value on time to last follow-up.

## Results

### Distribution of tumor variant and neoepitope burdens

**Fig. 1:**
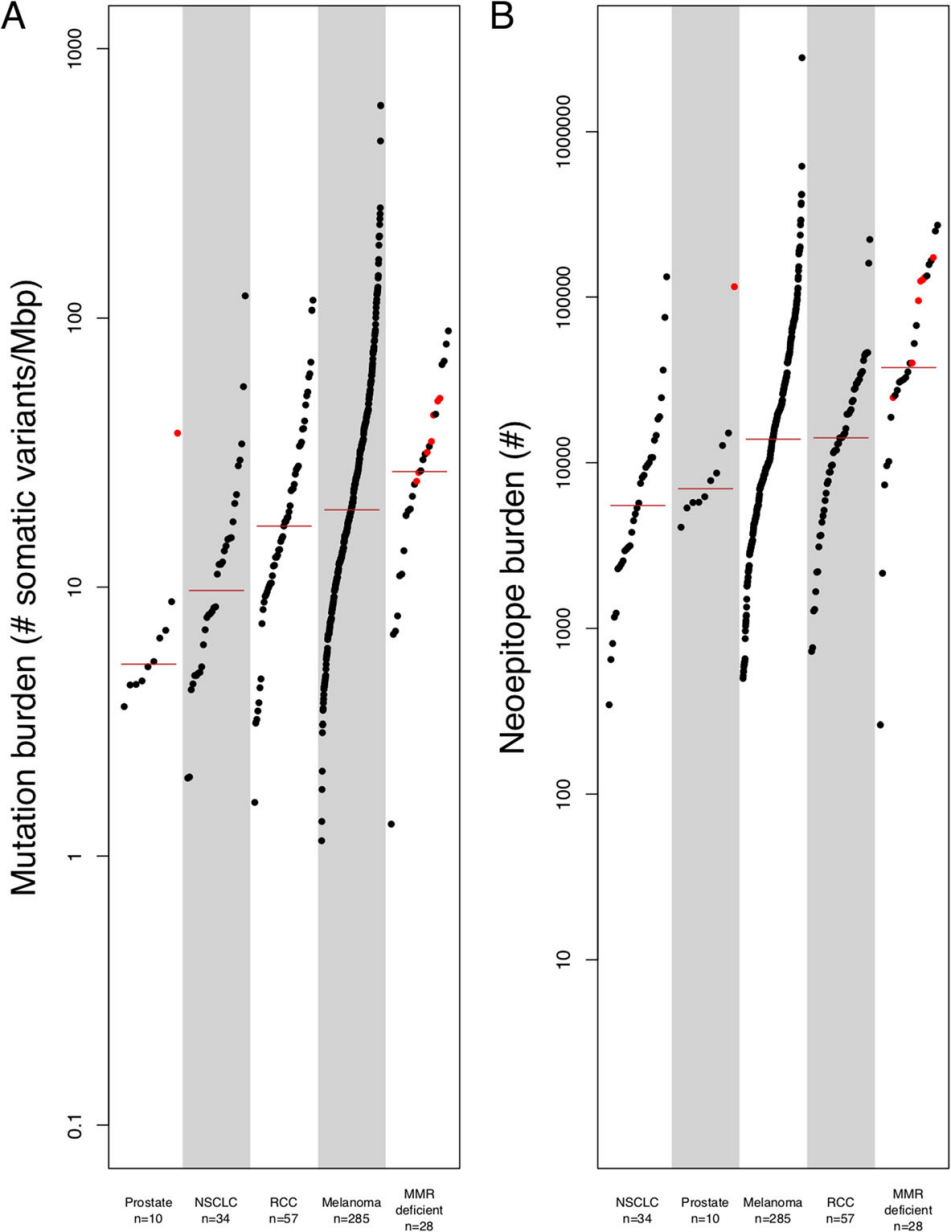
Per-patient distribution of mutation and neoepitope burdens across 7 cancer types. **a** The number of somatic DNA variants per patient (scaled for sequence coverage) are shown along the *y*-axis, with each dot representing an individual cancer patient (cancer types shown along the *x*-axis). Note that MMR-deficient cancers here represent a cohort of three different cancer types including colon, endometrial, and thyroid with evidence of mismatch repair deficiency as determined by polymerase chain reaction or immunohistochemistry [9]. Red colored dots correspond to patients with microsatellite instability as determined by mSINGS (see “Methods”). **b** The number of putative neoepitopes per patient are shown along the *y*-axis, with each dot representing an individual cancer patient (cancer types shown along the *x*-axis). Abbreviations as follows: MMR = mismatch repair

**Fig. 2:**
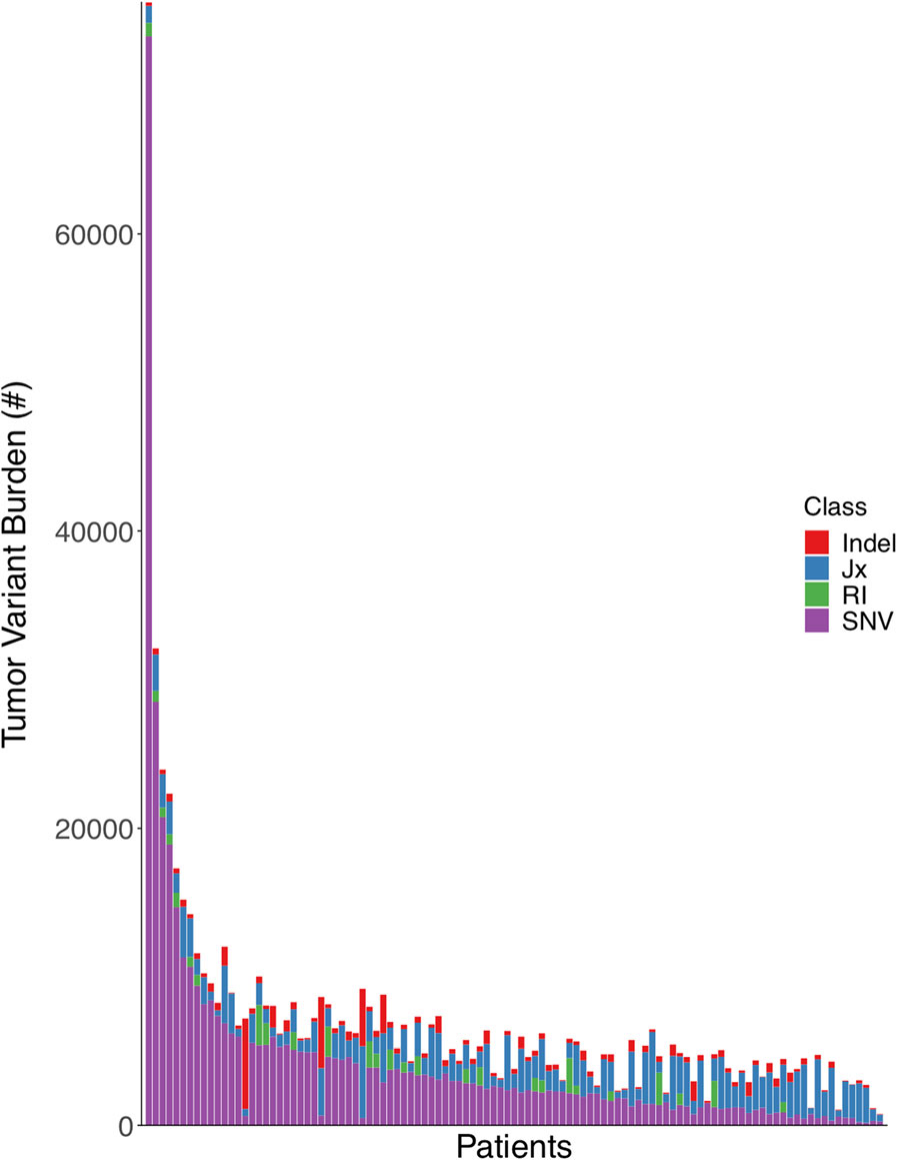
Per-patient distribution of overall tumor variant burden and its components. The number of total tumor variants per patient is shown along the *y*-axis, with the numbers of retained introns (RI), tumor-specific exon-exon junctions (Jx), insertions/deletions (Indel), and single nucleotide variants (SNV) shown in green, blue, red, and purple, respectively. The data for each individual patient is displayed as stacked bars along the *x*-axis, sorted from left to right by the number of single nucleotide variants (from highest to lowest)

We find that the median TMB (based on consensus DNA variant calls; see “Methods”) varies by an order of magnitude across disease types, ranging from 635.5 variants for prostate cancer to 5632.5 variants for MMR-deficient cancers (Additional file 2: Figure S2). Adjusting by genome coverage for each patient (see “Methods”), the median TMB was 18.03 mutations/Mbp of genomic coverage (ranging from 5.17 for prostate cancer to 26.82 for MMR-deficient cancers, see Fig. 1a). The majority of variants were found to be single nucleotide variants (median 85.07% per patient), with the remainder from in-frame and frameshift insertions and deletions (ranging from a median 7.52% indels for RCC to 37.26% indels for prostate cancer, see Additional file 2: Figure S3). Note that RNA variants such as alternative exon-exon junctions and retained introns were also assessed in the subset of patients with corresponding RNA-sequencing data (see “Methods”). Overall, tumor-specific junction burden appeared to be less variable across cancer types (ranging from 1301 for RCC to 2048.5 for melanoma). While retained introns (RI) have also been described as a source of neoepitopes [8], only 27 melanoma patients with RNA-seq data had any predicted RIs, with a median RI burden in those patients of 929 introns. Integrating these tumor DNA and RNA variants (given matched RNA-seq data) into a single combined tumor variant burden (TVB) yielded a median increase of 2345 variants per patient, with RNA sources of variation accounting for an average 40.8% of overall variants (see Fig. 2). Moreover, consideration of DNA variant burden alone neglects substantial somatic variation for some patients, as RNA sources of variation can constitute up to 86.7% of TVB.

As TMB and TVB are indirect assessments of cancer neoantigen load, we next calculated DNA-derived, RNA-derived, and overall neoepitope burdens per patient from putative protein-level variation (see “Methods”). The median per-patient DNA-derived neoepitope burden (for peptides predicted to bind to at least one of a patient’s MHC Class I or II alleles) was 13,512 peptides (ranging from 5511.5 for NSCLC to 37,710.5 for MMR-deficient cancers, see Fig. 1b) and was highly correlated with TMB itself (Pearson’s product-moment correlation of 0.63, *p* < 2.2 × 10^− 16^; see Additional file 2: Figure S4). There were generally more MHC Class I epitopes than Class II epitopes, with a median Class I epitope burden of 6337 peptides (ranging from 2366 for NSCLC to 15,645.5 for MMR-deficient cancers) and a median Class II epitope burden of 6027 peptides (ranging from 2167.5 for NSCLC to 23,554.5 for MMR-deficient cancers). We also assessed the burden of Class I epitopes predicted to be processed via the proteasome, transported through TAP, and presented on the cell surface (see “Methods”), with a median 771 such epitopes per patient (ranging from 366 for prostate cancer and 2536.5 for MMR-deficient cancers). While not all patients possessed RIs, the median per-patient RNA-derived neoepitope burden among the 27 melanoma patients with predicted RIs (366,843 peptides) was an order of magnitude higher than DNA-derived neoepitopes in the vast majority of cases (Additional file 2: Figure S5).

**Fig. 3:**
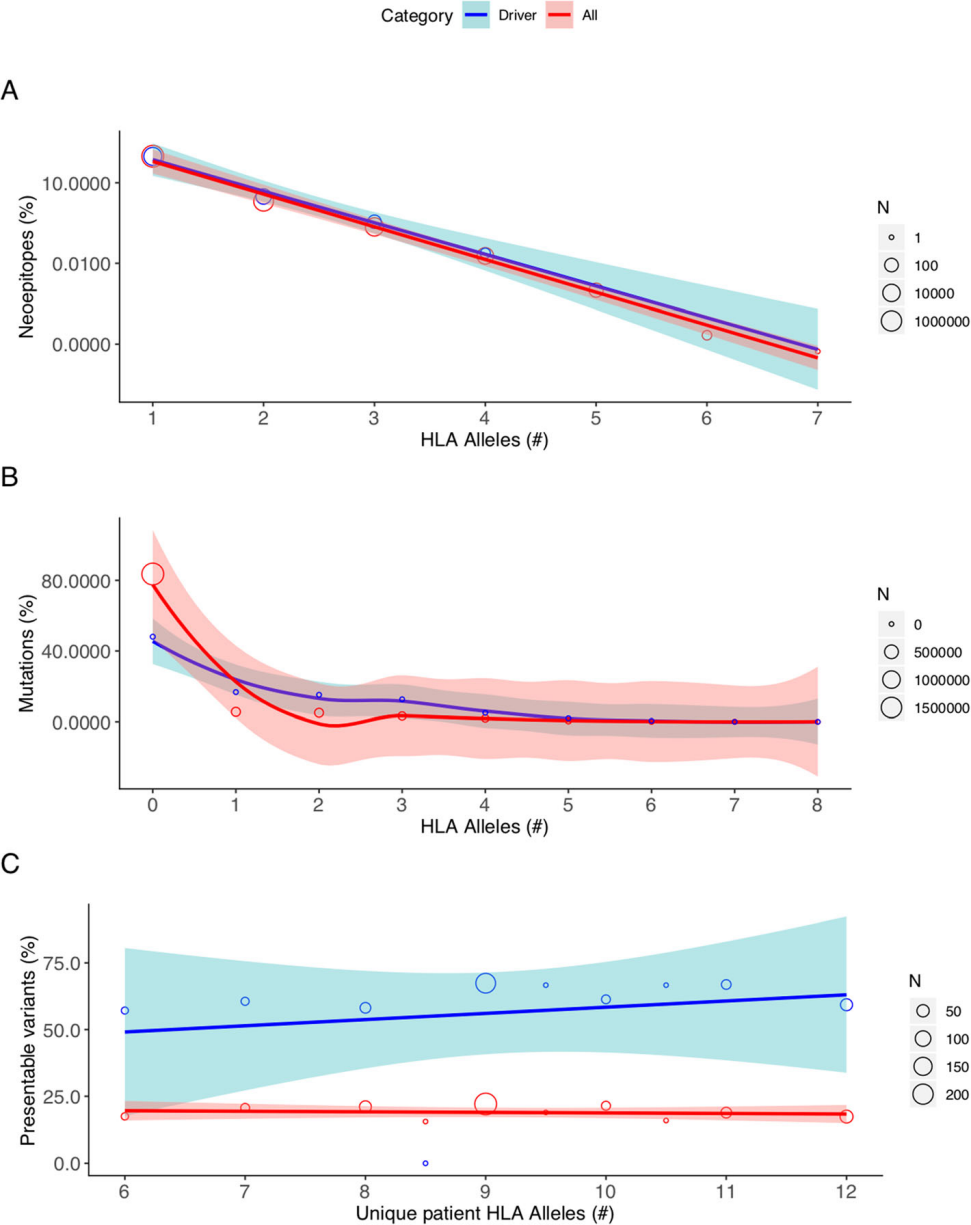
Robustness of putative neoepitope presentation. **a** The number of unique patient-matched HLA alleles that are predicted to present an individual neoepitope is shown along the *x*-axis, with the *y*-axis (log scale) corresponding to the overall percent of neoepitopes sharing that same robustness of HLA presentation. Red and blue curves denote the best fit line based on linear regression for all neoepitopes and those resulting from cancer driver mutations, respectively. The surrounding red and light blue shading denotes the 95% confidence interval for all and driver-derived neoepitopes, respectively. Individual data points are shown as open circles, whose diameter corresponds to the number of neoepitopes as shown by the corresponding scale at right. **b** The total number of unique patient-matched HLA alleles that are predicted to present one or more neoepitopes arising from a single DNA mutation is shown along the *x*-axis, with the *y*-axis corresponding to the overall percent of mutations sharing that same robustness of HLA presentation. Red and blue curves denote the best fit line based on local polynomial regression for all mutations and cancer driver mutations, respectively. The surrounding red and light blue shading denotes the 95% confidence interval for all and driver mutations, respectively. Individual data points are shown as open circles, whose diameter corresponds to the number of mutations as shown by the corresponding scale at right. **c** The percentage of total variants that are predicted to be presented by one or more patient-matched HLA alleles is shown along the *y*-axis, with the *x*-axis corresponding to the number of unique HLA alleles for that patient. Red and blue curves denote the best fit line based on linear regression for all mutations and cancer driver mutations, respectively. The surrounding red and light blue shading denotes the 95% confidence interval for all and driver mutations, respectively. Individual data points are shown as open circles, whose diameter corresponds to the number of mutations as shown by the corresponding scale at right. Note that a predicted HLA binding affinity threshold of ≤ 500 nM was used in all cases (see “Methods”)

In addition to reporting the bulk number of neoepitopes per patient, we also analyzed the distribution of peptide presentation by patient-specific HLA types. Overall, a median of 8.91% of possible peptides are presented by one or more patient-specific MHC Class I or II alleles. Among these, any given neoepitope is, on average, only presented by a single MHC allele (Fig. 3a, Additional file 2: Figure S6A). There are many additional degrees of freedom to surveil the peptide-level consequences of an individual variant (e.g., individual single nucleotide variants may give rise to as many as 272 different peptides of 8-24aa lengths, any of which might be presented via one or more MHC Class I or II alleles). As such, we find that 83.4% of all DNA variants resulting in peptide-level change(s) have at least one neoepitope putatively presented by at least one HLA allele, with a median of three different HLA alleles able to present one or more neoepitopes from each individual variant (Fig. 3b, Additional file 2: Figure S6B). Moreover, the percentage of variants presented increases with increasing MHC heterozygosity (Fig. 3c, Additional file 2: Figure S7). Within the cohort, 329 patients had pathogenic cancer-related mutations (see “Methods”), with an average of 2.8 such variants per patient among those patients. Consistent with prior work demonstrating a relative paucity of peptide presentation from cancer driver mutations [50], we find that a smaller number (approximately 68.5%) of driver variants in this cohort yielded neoepitopes, with only 10.4% of neopeptides from these variants on average being predicted to bind to any of a patient’s HLA alleles (Fig. 3).

### Tumor variant and neoepitope burdens as predictors of response and survival

We next sought to quantify immunotherapy response rate as a function of TMB, TVB, and neoepitope burden. Using disease-specific logistic regression models, we found that neither TVB nor neoepitope burden were significant predictors of immunotherapy response (see Table 1). In contrast, for NSCLC patients there was a 120.8% increase in the odds of response per log2 fold change in TMB (*p* = 0.034), though these results were not significant upon adjustment for multiple hypothesis testing (*p* = 0.053). Similar effects were not seen for melanoma (*p* = 0.267) or RCC (*p* = 0.973).

**Table 1:** Immunotherapy (αPD1 and αCTLA4) response probability based on logistic regression models of tumor mutational burden (TMB), neoepitope burden (Neoepitopes), and combined tumor DNA- and RNA- variant burden (TVB) for melanoma, non-small cell lung cancer (NSCLC), and renal cell carcinoma (RCC). *P* values are reported on a per-model basis without correction for multiple comparisons per cancer type

Cancer type & Metric	aPD1			aCTLA4			*p*-value
	N	Response Probability (25th %ile)	Response Probability (75th %ile)	N	Response Probability (25th %ile)	Response Probability (75th %ile)	
Melanoma							
TMB	50	0.456	0.507	195	0.298	0.342	0.267
Neoepitopes	50	0.470	0.507	195	0.302	0.334	0.378
TVB	27	0.367	0.490	61	0.307	0.424	0.079
NSCLC							
TMB	33	0.291	0.578				0.034
Neoepitopes	33	0.345	0.583				0.053
RCC							
TMB	50	0.656	0.666				0.894
Neoepitopes	50	0.662	0.66				0.973
TVB	17	0.641	0.538				0.600

**Fig. 4:**
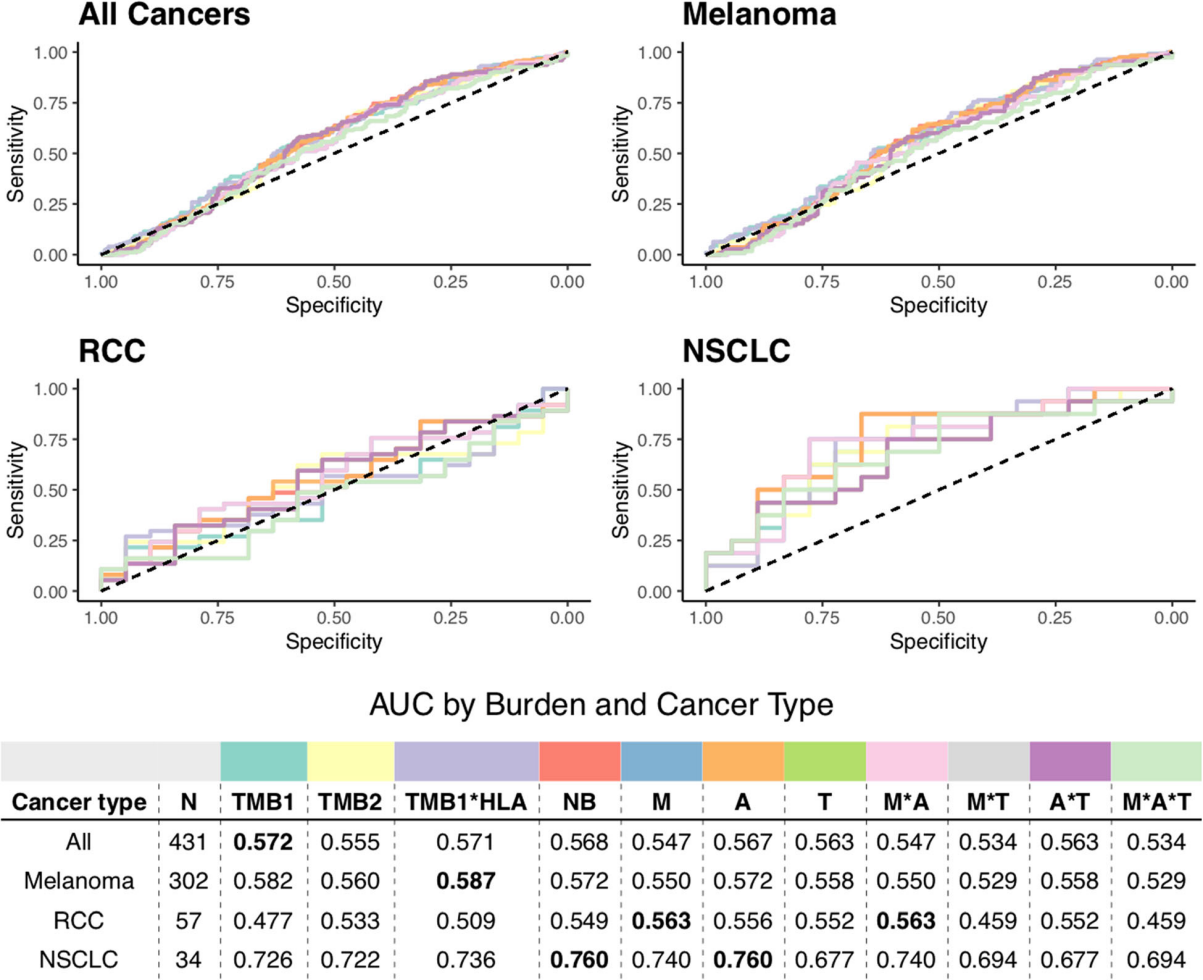
Receiver operating characteristic curves of predictive capacity of 11 different mutation/neoepitope burden metrics. The upper panels depict the true positive rate (sensitivity, *y*-axis) and false positive rate (1-specificity, *x*-axis) for each metric across all probability thresholds. The four panels represent models for four different cohorts based on different subsets of patients: All Cancers, which includes all patients, and Melanoma, RCC, and NSCLC, which include only melanoma, RCC, and NSCLC patients, respectively. The table in the lower panel reports the area under the curve (AUC) for each metric (columns) applied to a different cancer cohort (rows), with colors above the methods indicating the color of the corresponding curve in the upper panels. TMB is used as a predictor in both raw (TMB1) and coverage-adjusted (TMB2) forms, as well as in a multiplicative combination with patient HLA allele count (TMB1*HLA). Neoepitope burden (NB) is used as a predictor in both raw and extended formats (see “Methods”). Extended neoepitope burden metrics include number of amino acid mismatches (M), number of HLA alleles predicted to bind each epitope (A), and number of transcripts expressing each epitope in TCGA (T), along with their multiplicative combinations. Bold-faced values indicate the best value for each cancer cohort

Coverage-adjusted SNV burden predicted response to immunotherapy better than overall TMB or any indel burden for all cancer types except RCC, for which coverage-adjusted burden of in-frame indels was the most predictive burden (see Additional file 2: Figure S8). Neoepitope burden alone predicted response to immunotherapy comparatively well as TMB, calculated using both raw and coverage-adjusted counts (see Fig. 4). There was no difference in predictive capacity between Class I vs Class II epitope burdens (see Additional file 2: Figure S9). Similarly, incorporation of proteasomal cleavage, TAP transport, and cell surface presentation did not improve predictive capacity compared to TMB and neoepitope burden (see Additional file 2: Figure S10). We also weighted neoepitope burden by several criteria hypothesized to be related to increased immunogenicity, including number of amino acid mismatches per peptide, number of MHC alleles predicted to bind each peptide, and number of TCGA-expressed transcripts of origin for the peptide (see “Methods”). In all cases, these weighted burdens yielded similar predictive capabilities to TMB or unadjusted neoepitope burden, though mismatch- and mismatch-by-allele-weighted neoepitope burdens incrementally improved predictive capacity for RCC patients, and allele-weighted neoepitope burden incrementally improved predictive capacity for NSCLC patients (see Fig. 4). Interestingly, global assessment of HLA presentation (unique HLA allele count per patient) added slight predictive capacity to TMB in melanoma, RCC, and NSCLC patients (see Fig. 4). However, the capacity for any of these metrics to predict patient-level immunotherapy response varied substantially by cancer type, with the highest predictive power for the NSCLC cohort, but a very limited predictive capability in melanoma, RCC, or when pooled across all cancer types (see Fig. 4). Indeed, TMB as calculated by consensus variant calls predicts immunotherapy response more poorly than the experimental noise of the breadth of genomic coverage (Mbp) obtained via DNA sequencing in melanoma, RCC, and when pooled across cancer types (see Additional file 2: Figure S11).

**Fig. 5:**
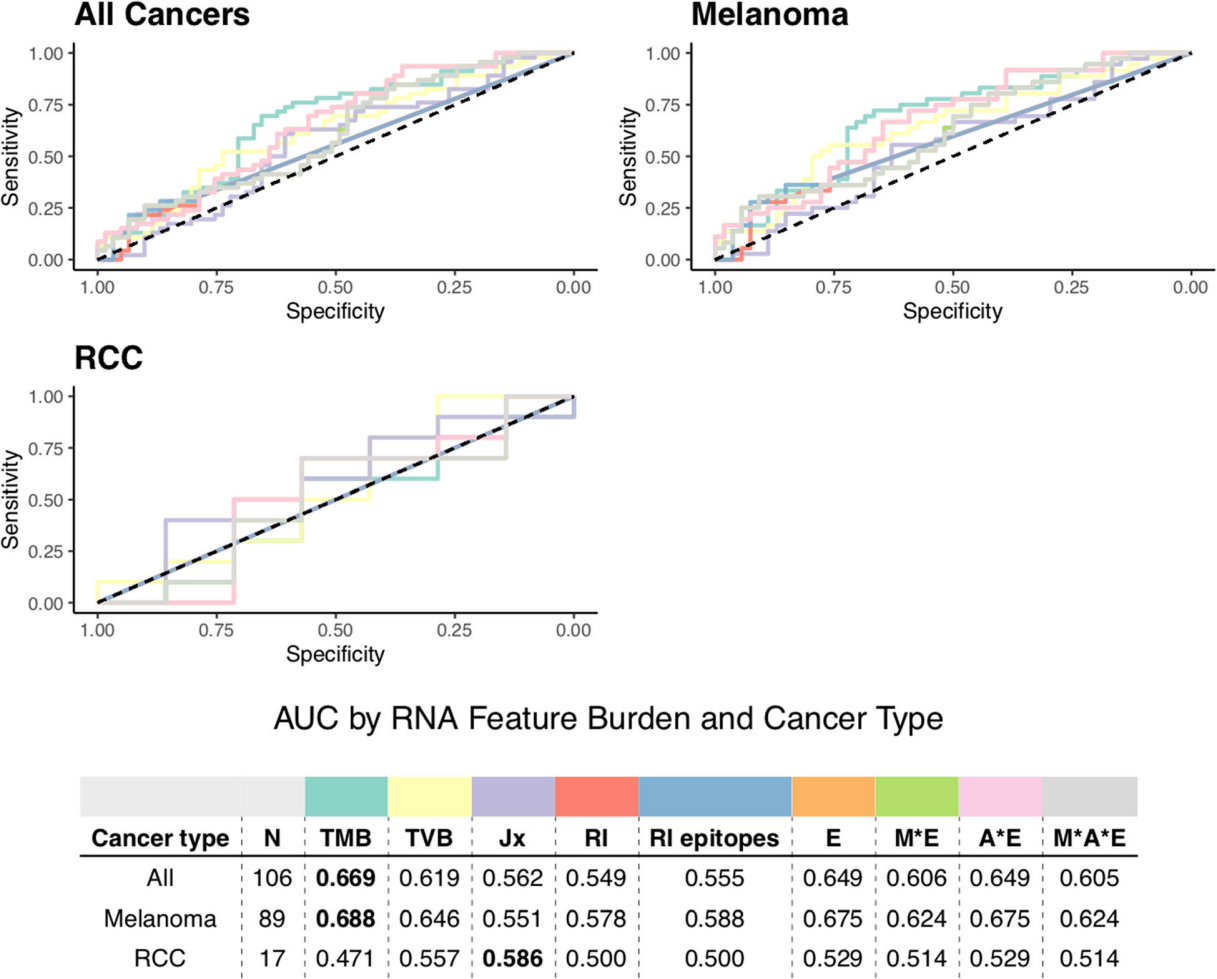
Receiver operating characteristic curves of predictive capacity of nine different variant/neoepitope burden metrics. The upper panels depict the true positive rate (sensitivity, *y*-axis) and false positive rate (1-specificity, *x*-axis) for each metric across all probability thresholds. The three panels represent models for three different cohorts based on different subsets of patients: All Cancers, which includes all patients, and Melanoma, and RCC, which include only melanoma and RCC patients, respectively. The table in the lower panel reports the area under the curve (AUC) for each metric (columns) applied to a different cancer cohort (rows), with colors above the methods indicating the color of the corresponding curve in the upper panels. TMB and TVB are used as predictors in the raw formats. Jx represents the number of tumor-specific junctions per patient, and RI represents the number of retained introns per patient, with RI epitopes representing neoepitopes derived from those retained introns. Neoepitope burden is used as predictor in its RNA-feature-extended formats (see “Methods”). Extended neoepitope burden metrics include number of expressed transcripts for each epitope (E), number of amino acid mismatches (M), number of HLA alleles predicted to bind each epitope (A), and number of transcripts expressing each epitope in TCGA (T), along with their multiplicative combinations

For patients with tumor RNA-seq data, we also investigated how TVB and RNA-derived neoepitopes predicted response to immunotherapy (see Fig. 5). We specifically considered tumor-specific junction burden, retained intron burden, retained intron neoepitope burden, and patient-specific expression-weighted neoepitope burdens (see “Methods”). As before, the vast majority of metrics (e.g., TMB, TVB) were all comparable in terms of predictive performance. However, considering these RNA-derived features did not increase predictive capacity over TMB, with the exception of the burden of tumor-specific splicing junctions, which yielded an increase in predictive performance for RCC patients (see Fig. 5).

**Fig. 6:**
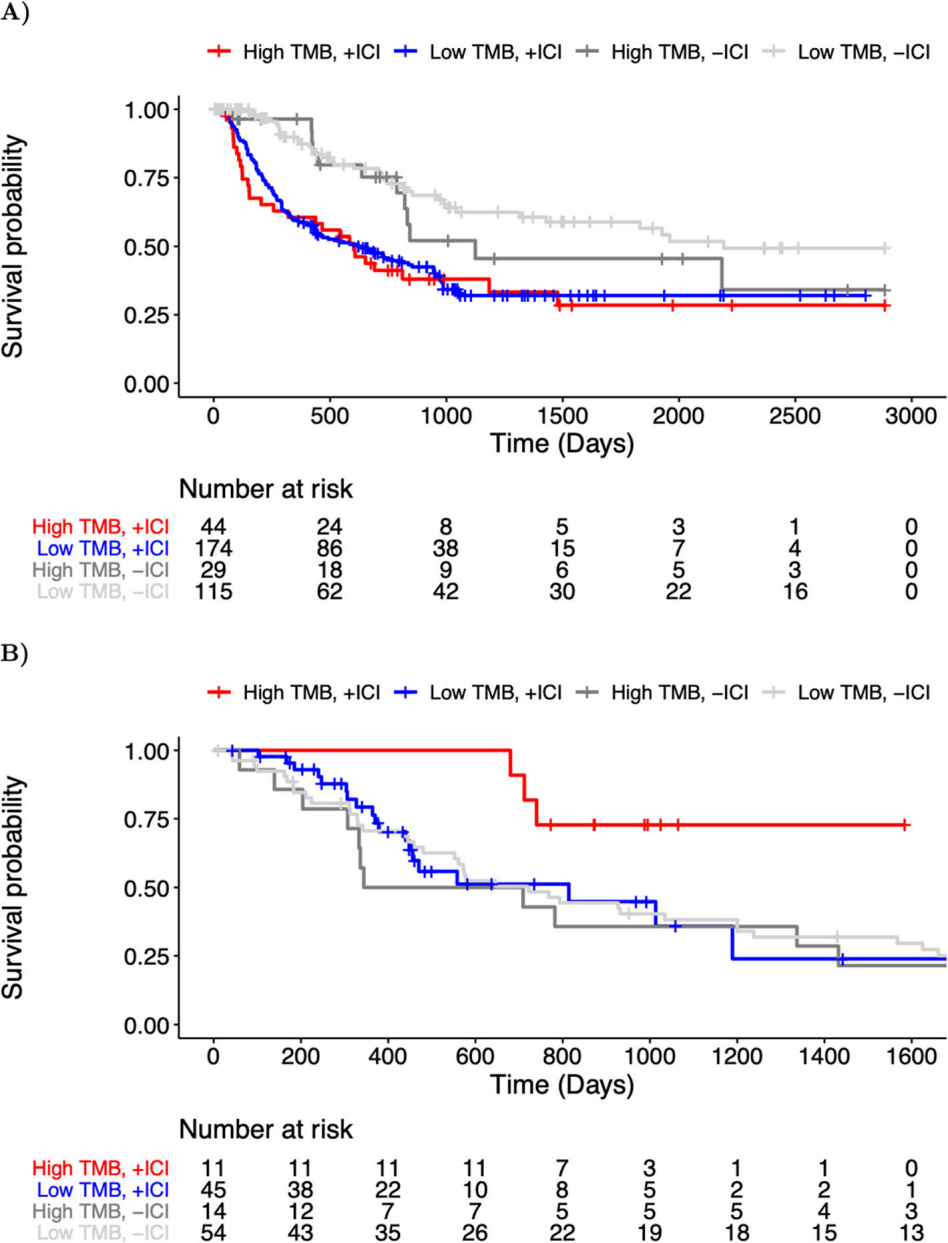
Overall survival among cancer patients with high and low TMB. **a** Kaplan-Meier curves for immunotherapy-treated (+ICI) and immunotherapy-naive (−ICI) Stage III-IV melanoma patients with high TMB (> 80th percentile) are shown in red, and dark gray, respectively, while immunotherapy-treated (+ICI) and immunotherapy-naive (−ICI) patients with low TMB (≤ 80th percentile) are shown in blue and light gray, respectively. The underlying table corresponds to the number of patients at risk of death at each timepoint. Note: TCGA SKCM patient data (−ICI) is censored at 2885 days (maximal follow-up in immunotherapy-treated cohort) to emphasize comparable time-scales. **b** Kaplan-Meier curves for the immunotherapy-treated (+ICI) and immunotherapy-naive (−ICI) metastatic (Stage IV) renal cell carcinoma patients with high TMB (> 80th percentile) are shown in red, and dark gray, respectively, while immunotherapy-treated (+ICI) and immunotherapy-naive (−ICI) patients with low TMB (≤ 80th percentile) are shown in blue and light gray, respectively. The underlying table corresponds to the number of patients at risk of death at each timepoint. Note: TCGA KIRC patient data is censored at 1724 days (maximal follow-up in immunotherapy-treated cohort) to emphasize comparable time-scales

Using an established threshold for identifying tumors with “high” TMB, namely TMB that exceeds the disease-matched 80th percentile [2], we investigated the metric’s capacity to predict overall survival in the context of immune checkpoint blockade therapy. While not statistically significant (*p* > 0.05, based on Cox proportional hazard modeling), we saw a clear trend toward improved overall survival among individuals with renal cell carcinoma and a high TMB (Fig. 6a). Additionally, model comparisons using different TMB percentile cutoffs suggest that differences in overall survival for high and low TMB groups may be threshold dependent and alter model significance (see Additional file 2: Figure S12). Notably, the lack of a significant TMB effect may be due to insufficient sample size as the number of patients qualifying as high TMB decreases steadily with increasing threshold. In contrast, the same trend is not seen between TMB and overall survival among a separate cohort of patients (TCGA) in the absence of immunotherapy (Fig. 6a). We also observed no differences in survival among individuals with metastatic melanoma (Fig. 6b). In both cases, TVB and neoepitope burden demonstrate comparable capacities to stratify overall survival as TMB (Additional file 2: Figures S13 and S14).

### Metric instability of tumor variant and neoepitope burdens

We find, however, that TMB is not robust across variant calling methods. TMB as reported by individual variant calling tools was moderately similar to that reported by consensus calls (see Additional file 2: Figure S15), but variability in per-caller TMB increased with increasing number of variants (see Additional file 2: Figure S16). Additionally, the difference in TMB between the highest and lowest counts from individual callers per patient (median difference of 1840 variants per patient) reflects a substantial fraction of the overall TMB, accounting for a median 59.3% of the value in the metric overall (see “Methods”).

We also compared tumor mutational burden as reported by the authors of the original manuscripts from which our cohort originated with our standardized consensus approach. While author-reported and consensus values for TMB were significantly correlated (Pearson’s product-moment correlation of 0.35, *p* = 1.99 × 10^− 7^; see Additional file 2: Figure S17), we note that author-reported values have a universally higher predictive capacity than we observe using consensus data (Additional file 2: Figure S18). We also find important discrepancies in per-patient classification. Approximately 26.2% of patients are incongruously determined to be TMB “high” or “low” (using a TMB threshold > 80th percentile as per [2]); however, as many as 42.23% of patients may be dubiously classified using alternative thresholds (e.g., 29th–65th percentiles; see Additional file 2: Figure S19). Consensus and author-reported nonsynonymous mutation burdens exhibited a similar extent of correlation as well as per-patient instability of classification (Pearson’s product-moment correlation of 0.58, *p* < 2.2 × 10^− 16^; see Additional file 2: Figures S18 and S20). The correlation between consensus-derived neoepitope burden and that reported by the original manuscripts was weak and not statistically significant (Pearson’s product-moment correlation of 0.026, *p* = 0.70; see Additional file 2: Figure S21). Moreover, we find that response hazard ratios are not stable based on TMB thresholds, a phenomenon especially dramatic in the RCC cohort (see Additional file 2: Figure S12), and consistent with prior findings that a single TMB threshold is inappropriate to apply across different cancer types [2].

**Fig. 7:**
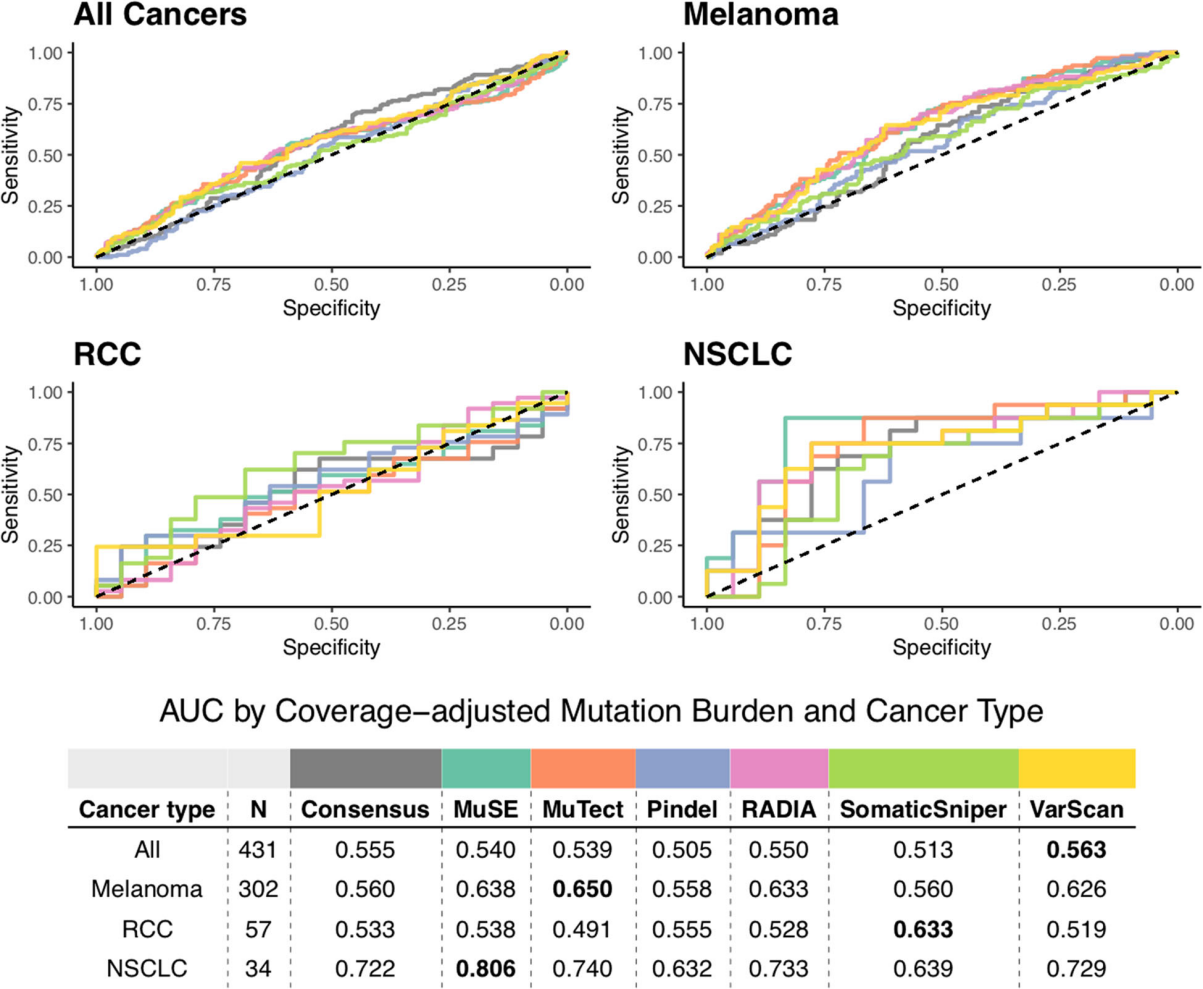
Receiver operating characteristic curves of predictive capacity of coverage-adjusted TMB from 7 different variant calling methods: consensus calling (see Methods), MuSE [51], MuTect [52], Pindel [53], RADIA [54], SomaticSniper [22], and VarScan 2 [23]. The upper panels depict the true positive rate (sensitivity, y-axis) and false positive rate (1-specificity, x-axis) for each method across all probability thresholds. The four panels represent models for four different cohorts based on different subsets of patients: All Cancers, which includes all patients, and Melanoma, RCC, and NSCLC, which include only melanoma, RCC, and NSCLC, respectively. The table in the lower panel reports the area under the curve (AUC) for each method (columns) applied to a different cancer cohort (rows), with colors above the methods indicating the color of the corresponding curve in the upper panels. TMB as determined by consensus calling (see “Methods”) is compared to the individual variant calling tools used in consensus calling. RCC = renal cell carcinoma, NSCLC = non-small cell lung cancer

Finally, we find that the predictive performance of TMB is sensitive to the method(s) used to perform variant calling (see Fig. 7). Note that the same phenomenon holds true for raw TMB counts (see Additional file 2: Figure S22). While outside the scope of the current manuscript, note also that the identity of resulting neoepitopes is also highly sensitive to the variant calling method (see Additional file 2: Figure S23).

## Discussion

To the best of our knowledge, this is the first study to evaluate TMB and correlated downstream metrics such as neoepitope burden from whole exome sequencing data using a gold standard ensemble approach [55, 56] applied to a meta-cohort of immunotherapy-treated cancer patients across multiple studies and disease types. This study also introduces the concept of tumor variant burden, incorporating potential RNA-derived sources of variants where available, and is the first study to estimate immunotherapy response rate as a function of TMB, TVB, and neoepitope burden. Moreover, this study is the first to quantitatively evaluate the stability of TMB as a metric, and the first to directly compare the predictive capacities of multiple TMB and related metrics.

Ultimately, we show that TMB is a dubious predictor of immunotherapy response, with substantial caveats regarding: (1) predictive capacity differences among different cancer types, with RCC being no better than random chance, (2) sensitivity of TMB and downstream metrics to variant calling methodology, and (3) stability of TMB thresholds and their ability to classify patients in a population. This suggests that the prospective clinical utilization of TMB is likely subject to many of these same issues and may result in unintended harms, whether due to omission of therapy for individuals with “low” TMB who might nonetheless benefit, or due to increased risk of toxicity in a “high” TMB population subject to overuse of immunotherapy. Indeed, a recent study of metastatic melanoma patients [57] found significantly different burdens of nonsynonymous mutations between disease subgroups, but not between progressors/responders, highlighting the instability of this metric.

With rare exception, we find no added predictive benefit to evaluating more complex bulk metrics downstream of TMB. Akin to prior observations, incorporation of HLA genotype diversity adds slightly to the predictive capacity of TMB [58]. Given the added technical effort and costs required to perform these analyses, we conclude that TMB is likely the optimal bulk assessment of tumor variation among those tested, though inclusion of HLA diversity data may marginally improve estimates. However, such bulk measurements neglect the potential importance of individual cancer neoantigens, which recent evidence suggests may be the driving force behind response to cancer immunotherapy by eliciting tumor-antigen-specific T cell responses [59].

This study has several limitations. First, numerous sampling based assays have also been used to assess TMB (e.g., [2, 60, 61]); however, we did not evaluate these data in this study, instead focusing on whole exome sequencing data as the prevailing gold standard for accurate mutational assessment. Note that these targeted assays would not enable incorporation of HLA allelic diversity data into a predictive model. Note also that there is wide variability among TMB assay design, analysis, and performance, with the potential for overestimation of TMB when using gene-targeted assays [62]. Ultimately, along with the substantial variability among widely used targeted assays [11], and the futility of expecting universal adoption of a single technique, this study highlights the need for increased standardization of TMB interpretation, a subject of active pursuit by the TMB Harmonization Project [63]. Second, we did not compare TMB in this dataset with other potential predictors of immunotherapy response (e.g., based on gene expression [64] or copy number instability [65]); however, it is possible that TMB could be synergistic with such orthogonal metrics. Third, by virtue of the retrospective nature of these data and limited availability of whole exome sequencing cohorts, this study cannot be assumed to translate to emerging immunotherapies and instead is interpretable exclusively for αPD1 and αCTLA4 therapy.

While this study is consistent with multiple prior reports demonstrating the importance of TMB in predicting immunotherapy response (e.g., [2, 66]), the caveats raised herein are of high concern for the field overall. Our collective emphasis on TMB is understandable given its relative ease of quantification using various techniques; however, it is indeed a dubious and indirect predictor. Tumors with higher TMB have been hypothesized to have more neoantigens that can be recognized by the immune system in response to checkpoint inhibition, yet the data presented here and data previously published [2] support the use of substantially different “absolute” TMB thresholds for immunotherapy response prediction across different diseases. Further, evidence suggests that other genomic factors, such as tumor purity and clonal heterogeneity, may further modulate the relationship between TMB and immunotherapy response [57, 67]. This suggests an added layer of as-of-yet undefined complexity not captured in the current bulk metrics, and likely related to disease-specific biology.

## Conclusions

In conclusion, we find sufficient cause to suggest that the predictive clinical value of TMB should not be overstated or oversimplified. While it is readily quantified, TMB is at best a limited surrogate biomarker of immunotherapy response. The data confirms TMB as a reasonable predictor in non-small cell lung cancer, and a weak predictor in melanoma. The data does not support TMB in isolation as a predictive biomarker for RCC, though it may be feasibly combined with HLA allelic diversity to achieve marginal performance.

## Supplementary information

Additional file 1: **Table S1.** (summary of patient samples used for analysis), **Table S2.** (summary of patient clinical and DNA variant data), **Table S3.** (per-patient summary of driver variants and neoepitopes), **Table S4.** (modified neoepitope burdens), **Table S5.** (tumor-specific splice junction burdens), **Table S6.** (tumor-specific retained intron and retained intron epitope burdens), **Table S7.** (processed neoepitope burdens).

Additional file 2: **Figure S1.** (visual depiction of potential splice variants captured), **Figure S2.** (per-patient distribution of raw mutation burdens across 7 cancer types), **Figure S3.** (per-patient distribution of insertion and deletion (indel) burdens across 7 cancer types), **Figure S4.** (TMB correlates with neoepitope burden), **Figure S5.** (per-patient distribution of overall tumor neoepitope burden and its components), **Figure S6.** (robustness of putative neoepitope presentation among 5 different cancer groups), **Figure S7.** (robustness of putative neoepitope presentation), **Figure S8.** (receiver operating characteristic curves of predictive capacity of 5 different coverage-adjusted variant burden metrics), **Figure S9.** (receiver operating characteristic curves of predictive capacity of MHC Class I vs. MHC Class II neoepitope burdens), **Figure S10.** (receiver operating characteristic curves of predictive capacity of processed neoepitope burden), **Figure S11.** (receiver operating characteristic curves of predictive capacity of Mbp of genomic coverage), **Figure S12.** (variation in estimated hazard ratio based on TMB threshold selection), **Figure S13.** (overall survival among melanoma patients with high and low tumor variant burden), **Figure S14.** (overall survival among melanoma and renal cell carcinoma patients with high and low neoepitope burden), **Figure S15.** (pairwise differences in normalized total mutation burden as determined by 7 different computational approaches), **Figure S16.** (variation in somatic mutation count increases with increased TMB from consensus variant calls), **Figure S17.** (author-reported total mutation burden correlates with consensus TMB), **Figure S18.** (receiver operating characteristic curves of predictive capacity of author-reported mutation and neoepitope burdens), **Figure S19.** (cohort-level disagreement in classification of individual patients as TMB or neoepitope burden “high” v. “low”), **Figure S20.** (author-reported nonsynonymous mutation burden correlates with nonsynoymous variants from consensus calling), **Figure S21.** (author-reported neoepitope burden correlates with neoepitopes derived from variants from consensus calling), **Figure S22.** (receiver operating characteristic curves of predictive capacity of TMB from 7 different variant calling methods), **Figure S23.** (detailed comparison of the complete set of neopeptide sequences predictions from MuSE, Mutect, Pindel, RADIA, SomaticSniper, VarScan, and consensus variant calling).

## Data Availability

Data used in these analyses is available on the Sequence Read Archive under accessions PRJNA278450 [4], PRJNA293912 [3], PRJNA305077 [68], PRJNA306070 [6], PRJNA307199 [5], PRJNA312948 [5], PRJNA324705 [12], PRJNA343789 [5], PRJNA357321 [13], PRJNA369259 [14], PRJNA414014 [16], PRJNA420786 [18], and PRJNA82745 [1] and on the European Genome-Phenome Archive under the accession EGAD00001004352 [15]. Data from Le et al. [9] and Graff et al. [17] are available from those authors upon reasonable request. The results of our analyses of these data are available in Additional file 1: Tables S2-S7. We have created a GitHub repository with instructions for reproducing results [69].

## Abbreviations

αCTLA4: Anti-cytotoxic T-lymphocyte-associated protein 4
αPD1: Anti-programmed cell death protein 1
AUC: Area under the curve
COAD: Colon adenocarcinoma
GATK: Genome Analysis Toolkit
GTEx: Genotype Tissue Expression
HLA: Human leukocyte antigen
Indel: Insertion or deletion
KIRC: Kidney renal clear cell carcinoma
kma: Keep Me Around
LUAD: Lung adenocarcinoma
LUSC: Lung squamous cell carcinoma
MHC: Major histocompatiblity complex
MMR: Mismatch repair
NSCLC: Non-small cell lung cancer
PRAD: Prostate adenocarcinoma
RCC: Renal cell carcinoma
RI: Retained intron
RNA-seq: RNA sequencing
ROC: Receiver operating characteristic
SKCM: Skin cutaneous melanoma
SRA: Sequence Read Archive
TCGA: The Cancer Genome Atlas
THCA: Thyroid carcinoma
TMB: Tumor mutational burden
TPM: Transcripts per million
TVB: Tumor variant burden
UCEC: Uterine corpus endometrial carcinoma
VCF: Variant call format
WES: Whole exome sequencing
