# Supplementary material for: Burden of tumor mutations, neoepitopes, and other variants are weak predictors of cancer immunotherapy response and overall survival
Source: Genome Med. 2020 Mar 30;12:33. doi: 10.1186/s13073-020-00729-2 (PMC7106909; doi:10.1186/s13073-020-00729-2)
Supplement: Supplementary file 2 — Figure S1. (visual depiction of potential splice variants captured), Figure S2. (per-patient distribution of raw mutation burdens across 7 cancer types), Figure S3. (per-patient distribution of insertion and deletion (indel) burdens across 7 cancer types), Figure S4. (TMB correlates with neoepitope burden), Figure S5. (per-patient distribution of overall tumor neoepitope burden and its components), Figure S6. (robustness of putative neoepitope presentation among 5 different cancer groups), Figure S7. (robustness of putative neoepitope presentation), Figure S8. (receiver operating characteristic curves of predictive capacity of 5 different coverage-adjusted variant burden metrics), Figure S9. (receiver operating characteristic curves of predictive capacity of MHC Class I vs. MHC Class II neoepitope burdens), Figure S10. (receiver operating characteristic curves of predictive capacity of processed neoepitope burden), Figure S11. (receiver operating characteristic curves of predictive capacity of Mbp of genomic coverage), Figure S12. (variation in estimated hazard ratio based on TMB threshold selection), Figure S13. (overall survival among melanoma patients with high and low tumor variant burden), Figure S14. (overall survival among melanoma and renal cell carcinoma patients with high and low neoepitope burden), Figure S15. (pairwise differences in normalized total mutation burden as determined by 7 different computational approaches), Figure S16. (variation in somatic mutation count increases with increased TMB from consensus variant calls), Figure S17. (author-reported total mutation burden correlates with consensus TMB), Figure S18. (receiver operating characteristic curves of predictive capacity of author-reported mutation and neoepitope burdens), Figure S19. (cohort-level disagreement in classification of individual patients as TMB or neoepitope burden “high” v. “low”), Figure S20. (author-reported nonsynonymous mutation burden correlates with nonsynoymous variant [file 13073_2020_729_MOESM2_ESM.pdf]

ADDITIONAL FILE 2: SUPPLEMENTARY FIGURES S1-S23

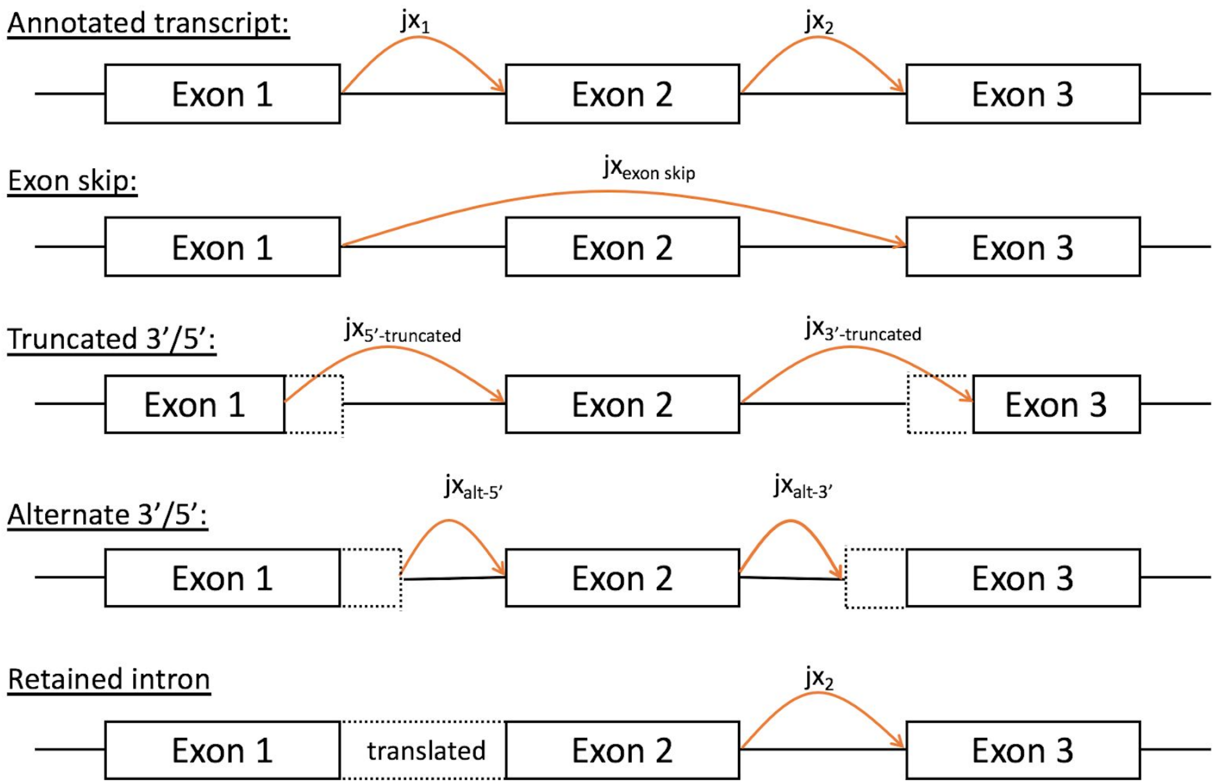

Supplementary Figure S1: *Visual depiction of potential splice variants captured. The top row shows the annotated "normal" splicing for a simulated gene with 3 exons; this splicing is represented by junctions (jxs) 1 and 2. A potential exon skip is represented on row 2, where exon 2 is skipped. Possible alternate 5' and 3' splice sites are shown in rows 3 and 4, and a retained intron between exons 1 and 2 in row 5.*

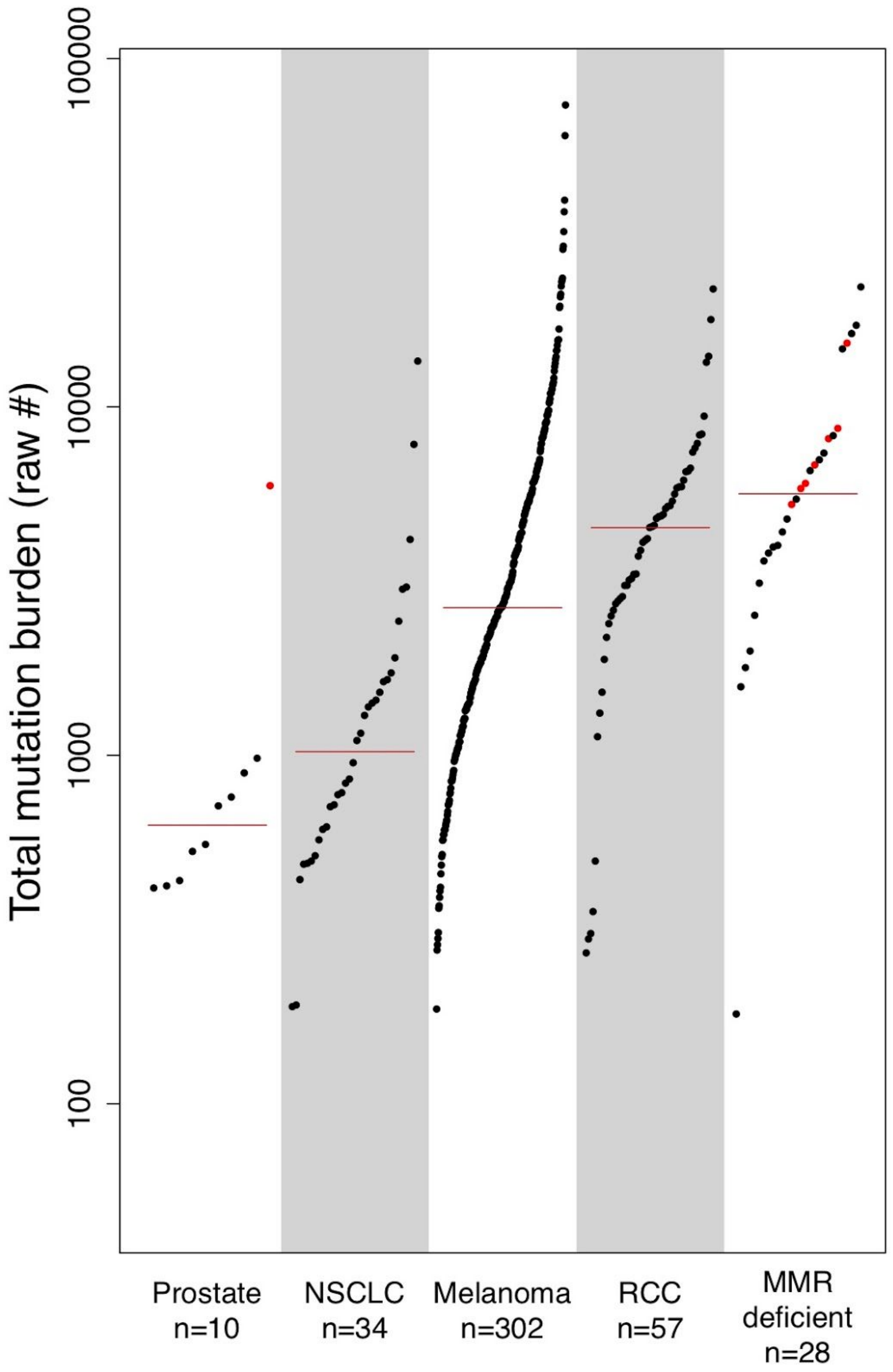

Supplementary Figure S2: *Per-patient distribution of raw mutation burdens across 7 cancer types. The raw number of somatic DNA variants per patient are shown along the y-axis, with each dot representing an individual cancer patient (cancer types shown along the x-axis). Note that MMR-deficient cancers here represent a cohort of 3 different cancer types including colon, endometrial, and thyroid with evidence of mismatch repair deficiency as determined by polymerase chain reaction or immunohistochemistry (9). Red colored dots correspond to patients with microsatellite instability as determined by mSINGS (see Methods). Abbreviations as follows: RCC=renal cell carcinoma, NSCLC=non-small cell lung cancer, MMR=mismatch repair.*

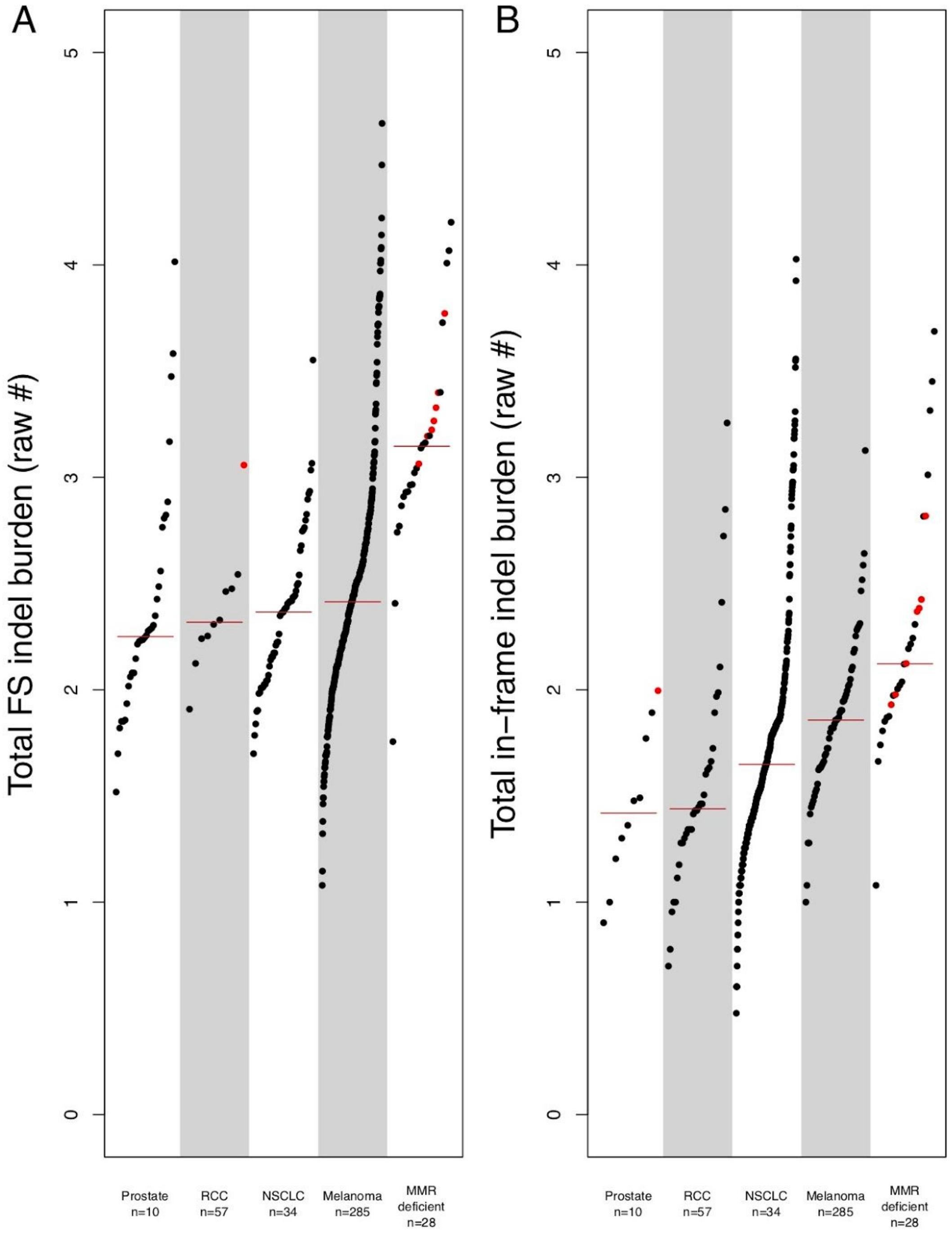

Supplementary Figure S3: *Per-patient distribution of insertion and deletion (indel) burdens across 7 cancer types. A) The number of somatic frameshift (FS) indels per patient are shown along the y-axis, with each dot representing an individual cancer patient (cancer types shown along the x-axis). Note that MMR-deficient cancers here represent a cohort of 3 different cancer types including colon, endometrial, and thyroid with evidence of mismatch repair deficiency as determined by polymerase chain reaction or immunohistochemistry (9). Red colored dots correspond to patients with microsatellite instability as determined by mSINGS (see Methods). B) The number of somatic in-frame indels per patient are shown along the y-axis, with each dot representing an individual cancer patient (cancer types shown along the x-axis). Abbreviations as follows: RCC=renal cell carcinoma, NSCLC=non-small cell lung cancer, MMR=mismatch repair.*

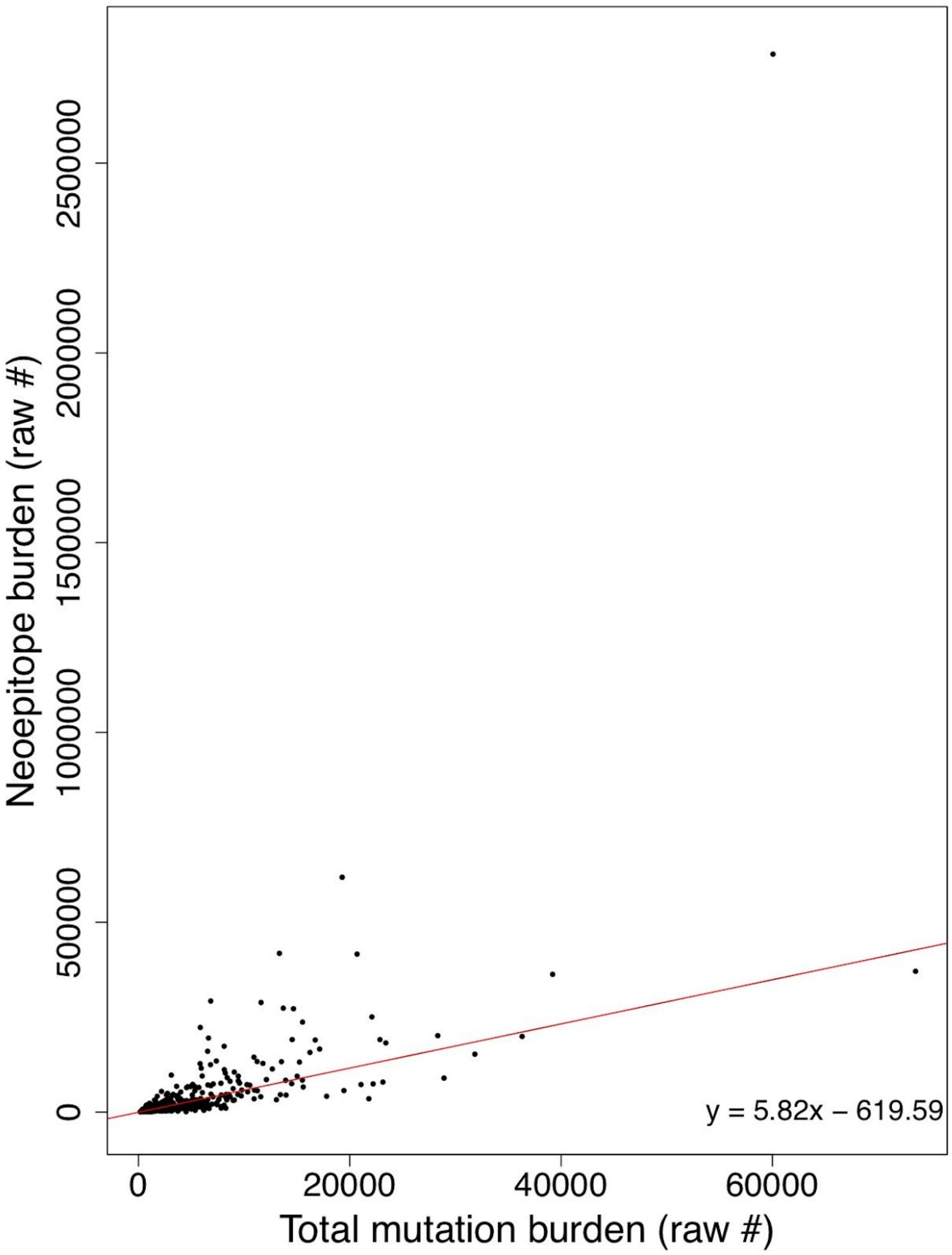

Supplementary Figure S4: *TMB correlates with neoepitope burden. Tumor mutational burden (x-axis) and neoepitope burden (y-axis) are strongly correlated (Pearson product-moment correlation of 0.63, $p < 2.2 \times 10^{-16}$). The best fit line as determined by linear regression is shown in red, with its equation in the bottom right corner.*

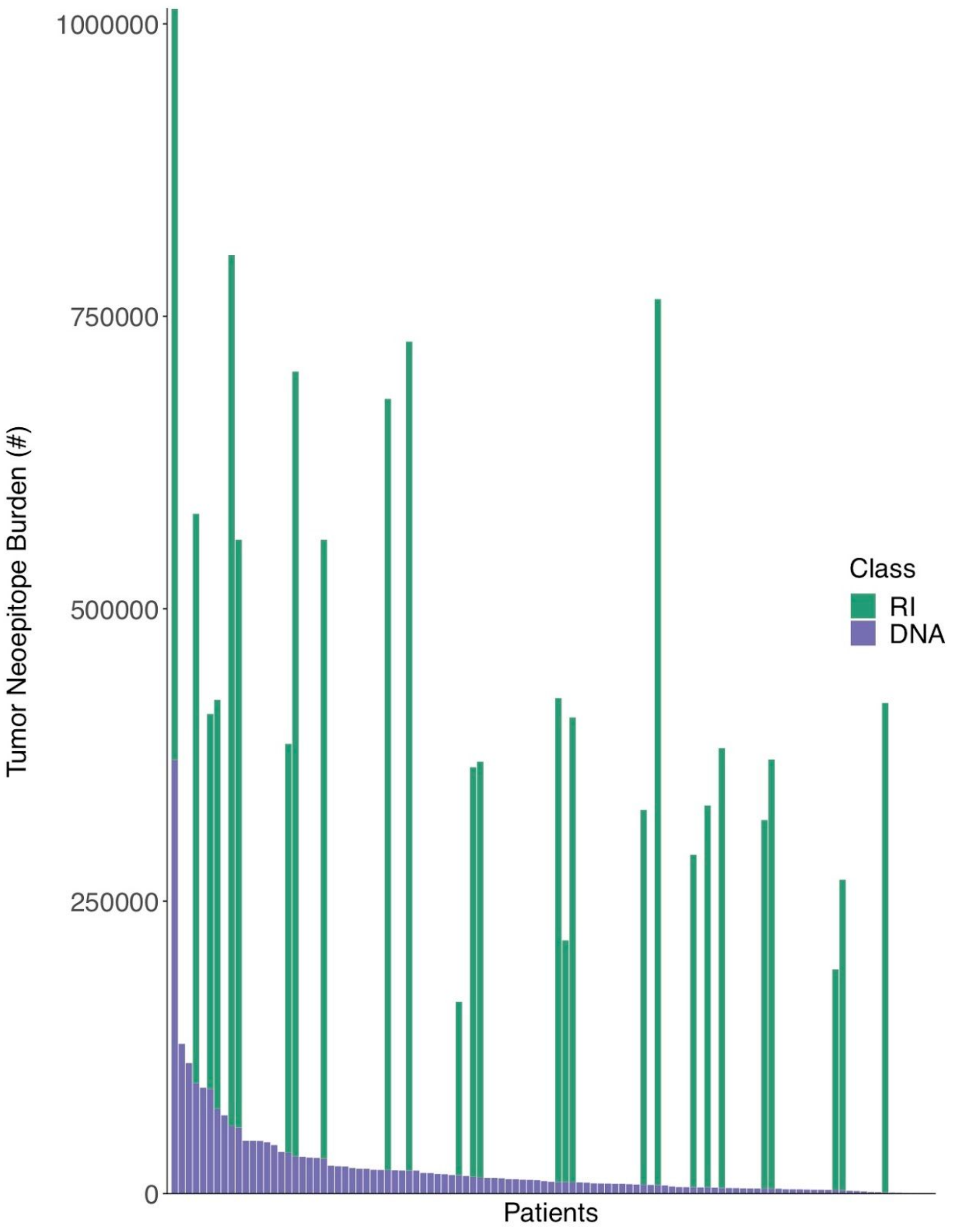

Supplementary Figure S5: *Per-patient distribution of overall tumor neoepitope burden and its components. The number of total tumor neoepitopes per patient is shown along the y-axis, with the numbers of neoepitopes derived from retained introns (RI) and somatic DNA variants (DNA) shown in green and purple, respectively. The data for each individual patient is displayed as stacked bars along the x-axis, sorted from left to right by the number of neoepitopes derived from somatic DNA variants (from highest to lowest).*

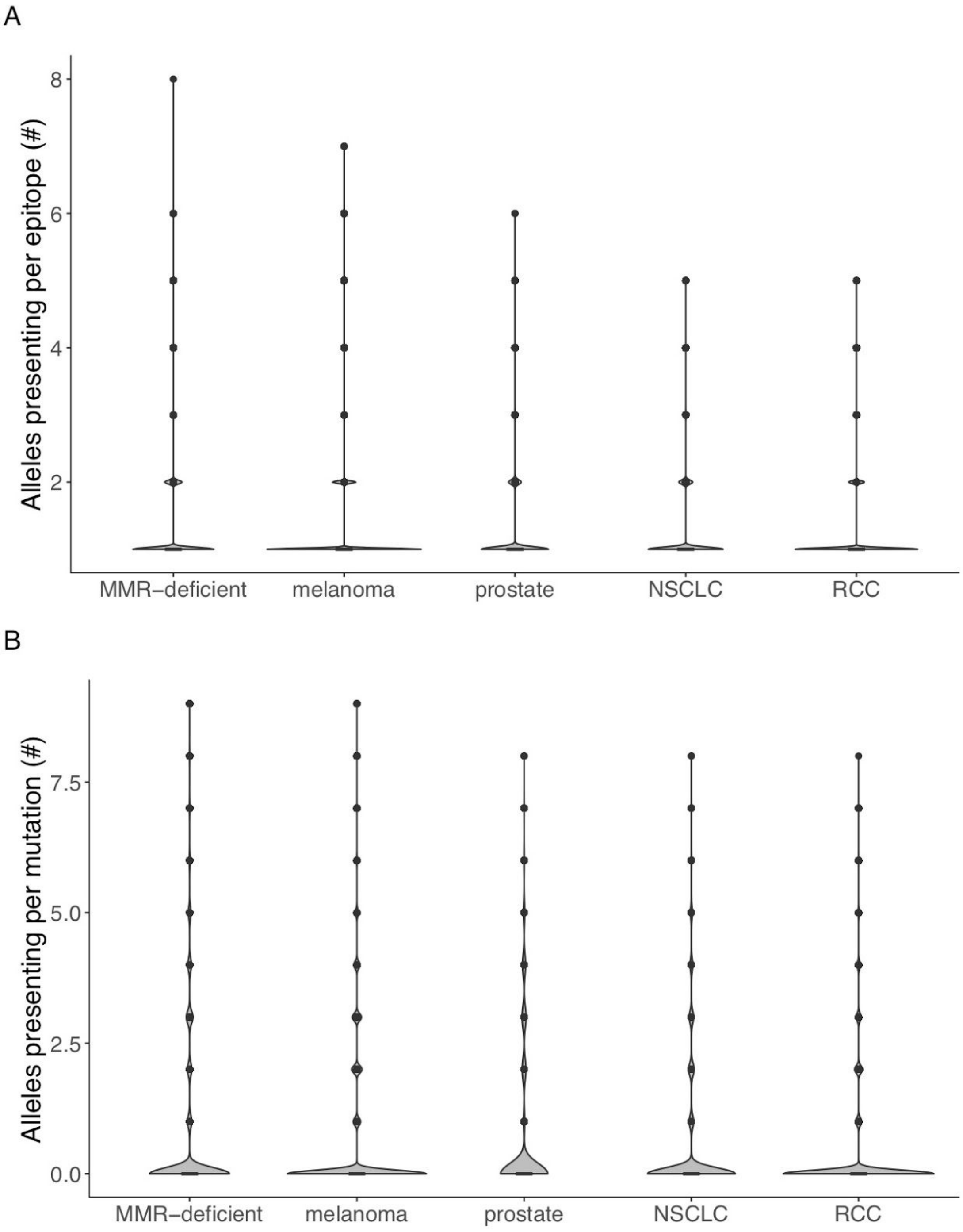

Supplementary Figure S6: *Robustness of putative neoepitope presentation among 5 different cancer groups. A) The number of unique patient-matched HLA alleles that are predicted to present an individual neoepitope is shown along the y-axis, with each violin plot distribution corresponding to a different cancer group along the x-axis, as labeled. Note that MMR-deficient cancers here represent a cohort of 3 different cancer types including colon, endometrial, and thyroid with evidence of mismatch repair deficiency as determined by polymerase chain reaction or immunohistochemistry (9). B) The total number of unique patient-matched HLA alleles that are predicted to present one or more neoepitopes arising from a single DNA mutation is shown along the y-axis, with each violin plot distribution corresponding to a different cancer group along the x-axis, as labeled. Note that the width of each violin plot at each point along the y-axis corresponds to the relative quantity of data points in that group for that value of the y-axis. Furthermore, the lower and upper borders of the box within each violin plot corresponds to the 25th and 75th percent quantiles of the dataset for that group, respectively, with the median value shown as a horizontal black line within the box. Note that a predicted HLA binding affinity threshold of ≤500nM was used in all cases (see Methods).*

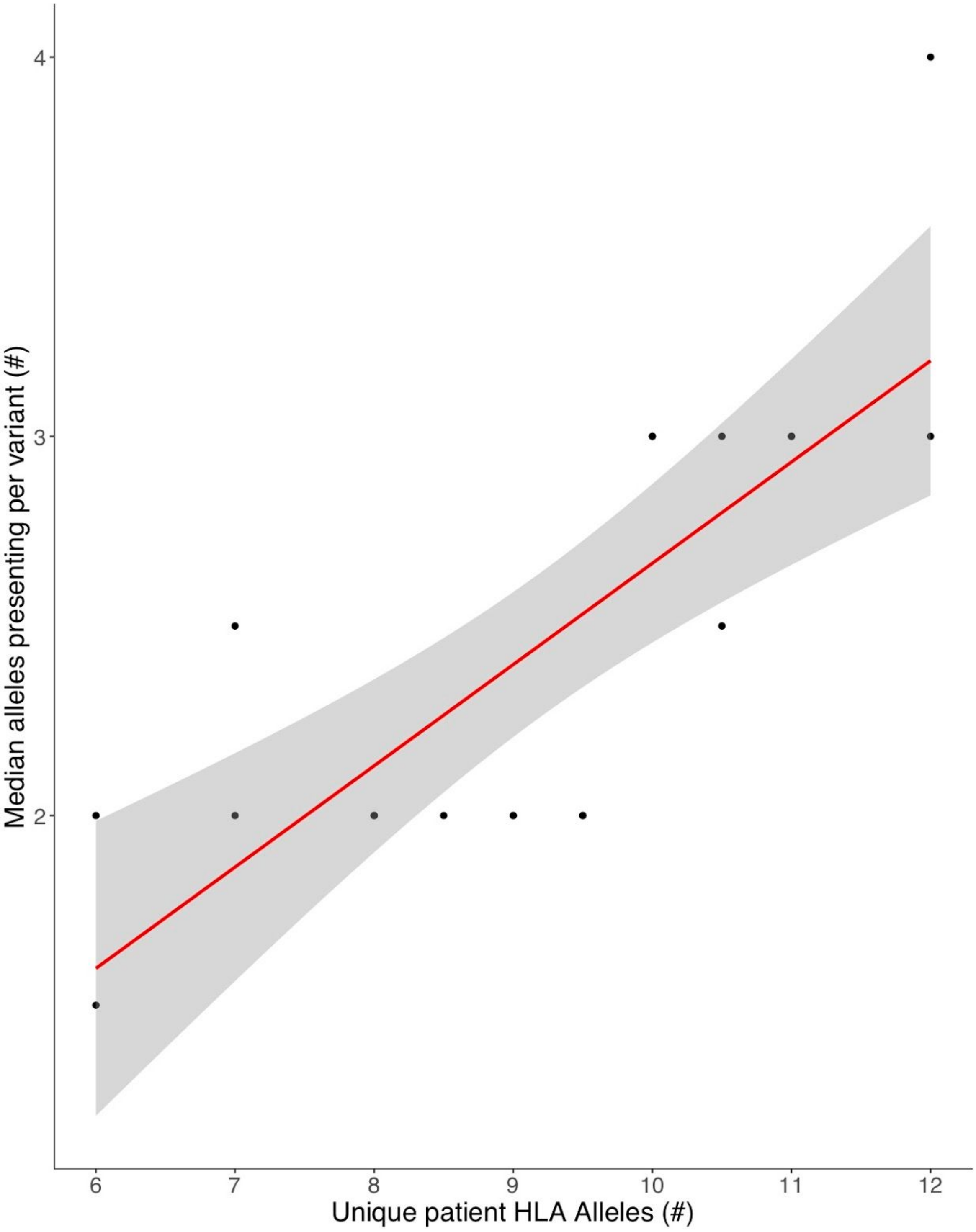

Supplementary Figure S7: *Robustness of putative neoepitope presentation. The median number of unique patient-matched HLA alleles that are predicted to present one or more neoepitopes arising from a single DNA mutation is shown along the y-axis, with the x-axis corresponding to patient-specific HLA heterozygosity (as the number of unique MHC I and II alleles per patient). Red curve denotes the best fit line based on linear regression, with surrounding gray shading denoting the 95% confidence interval. Note that a predicted HLA binding affinity threshold of ≤500nM was used in all cases (see Methods).*

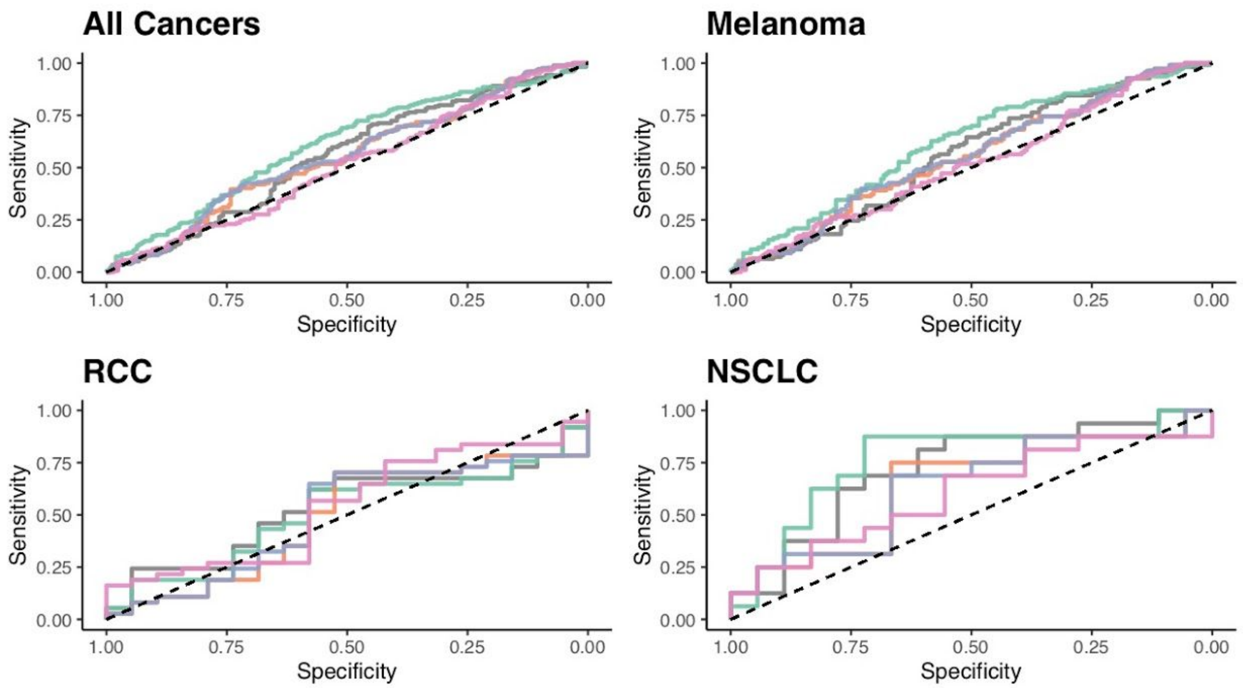

AUC by Mutation Burden and Cancer Type

Cancer type	N	All	SNVs	Indels	FS indels	In–frame indels
All	431	0.555	**0.609**	0.552	0.557	0.513
Melanoma	302	0.560	**0.619**	0.555	0.559	0.522
RCC	57	0.533	0.509	0.485	0.496	**0.542**
NSCLC	34	0.722	**0.760**	0.642	0.635	0.604

Supplementary Figure S8: *Receiver operating characteristic curves of predictive capacity of 5 different coverage-adjusted variant burden metrics. The upper panels depict the true positive rate (sensitivity, y-axis) and false positive rate (1-specificity, x-axis) for each metric across all probability thresholds. The three panels represent models for three different cohorts based on different subsets of patients: All Cancers, which includes all patients, and Melanoma, and RCC, which include only melanoma and RCC patients, respectively. The table in the lower panel reports the area-under-the-curve (AUC) for each metric (columns) applied to a different cancer cohort (rows), with colors above the methods indicating the color of the corresponding curve in the upper panels. All represents all DNA variants (SNVs and indels of all types), SNVs includes all single nucleotide variants, Indels includes all insertion/deletion variants, FS indels includes all frameshifting insertions and deletions, and In-frame indels includes all in-frame insertions and deletions.*

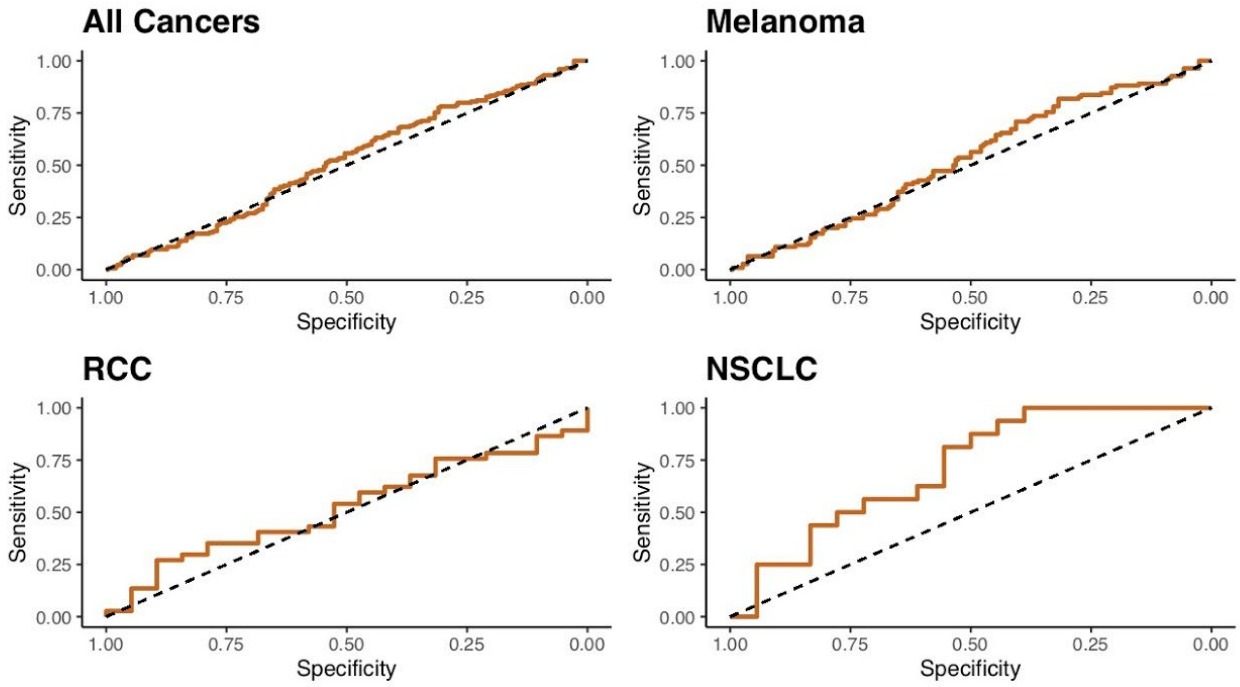

AUC by Neoepitope Burden and Cancer Type

Cancer Type	N	MHC Class I Epitopes	MHC Class II Epitopes
All	431	0.519	0.519
Melanoma	302	0.531	0.531
RCC	57	0.523	0.523
NSCLC	34	0.712	0.712

Supplementary Figure S9: *Receiver operating characteristic curves of predictive capacity of MHC Class I vs. MHC Class II neoepitope burdens. The upper panels depict the true positive rate (sensitivity, y-axis) and false positive rate (1-specificity, x-axis) for each metric across all probability thresholds. The three panels represent models for three different cohorts based on different subsets of patients: All Cancers, which includes all patients, and Melanoma, and RCC, which include only melanoma and RCC patients, respectively. The table in the lower panel reports the area-under-the-curve (AUC) for each metric (columns) applied to a different cancer cohort (rows), with colors above the methods indicating the color of the corresponding curve in the upper panels.*

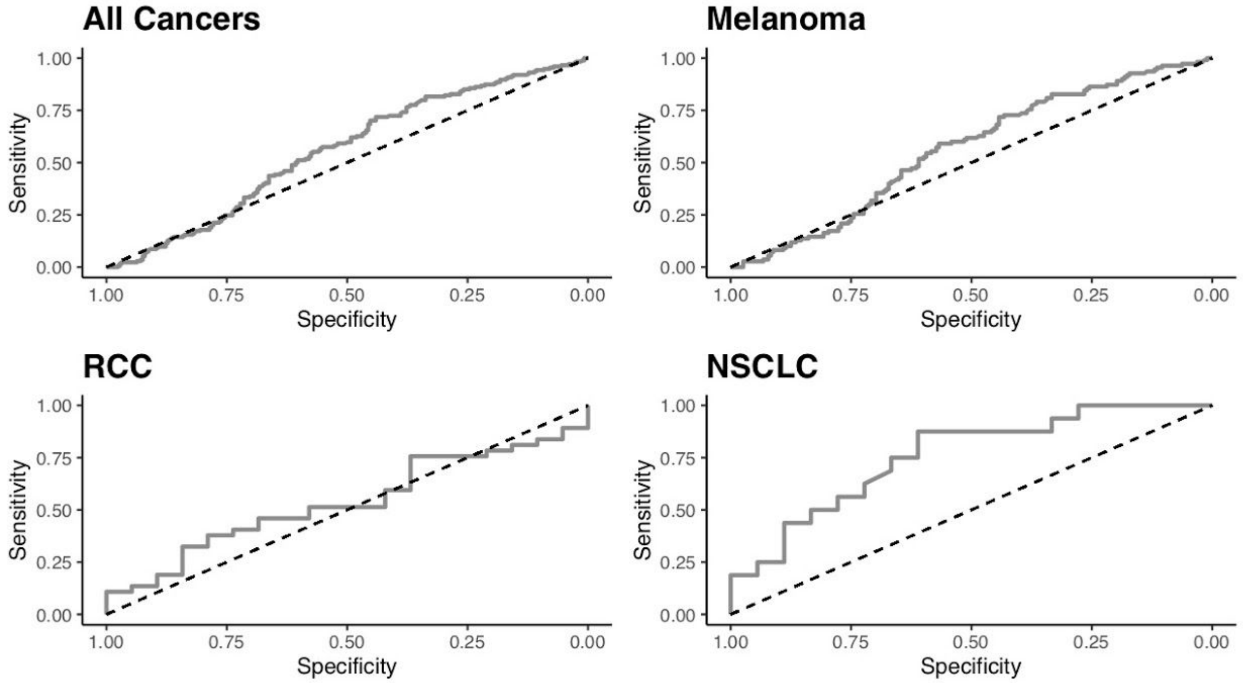

AUC by Processed Epitopes and Cancer Type

Cancer type	N	Processed epitopes
All	431	0.559
Melanoma	302	0.562
RCC	57	0.536
NSCLC	34	0.759

Supplementary Figure S10: *Receiver operating characteristic curves of predictive capacity of processed neoepitope burden. The upper panels depict the true positive rate (sensitivity, y-axis) and false positive rate (1-specificity, x-axis) for genomic coverage across all probability thresholds. The four panels represent models for four different cohorts based on different subsets of patients: All Cancers, which includes all patients, and Melanoma, RCC, and NSCLC, which include only melanoma, RCC, and NSCLC patients, respectively. The table in the lower panel reports the area-under-the-curve (AUC) for coverage (right column) applied to a different cancer cohort (rows). RCC=renal cell carcinoma, NSCLC=non-small cell lung cancer.*

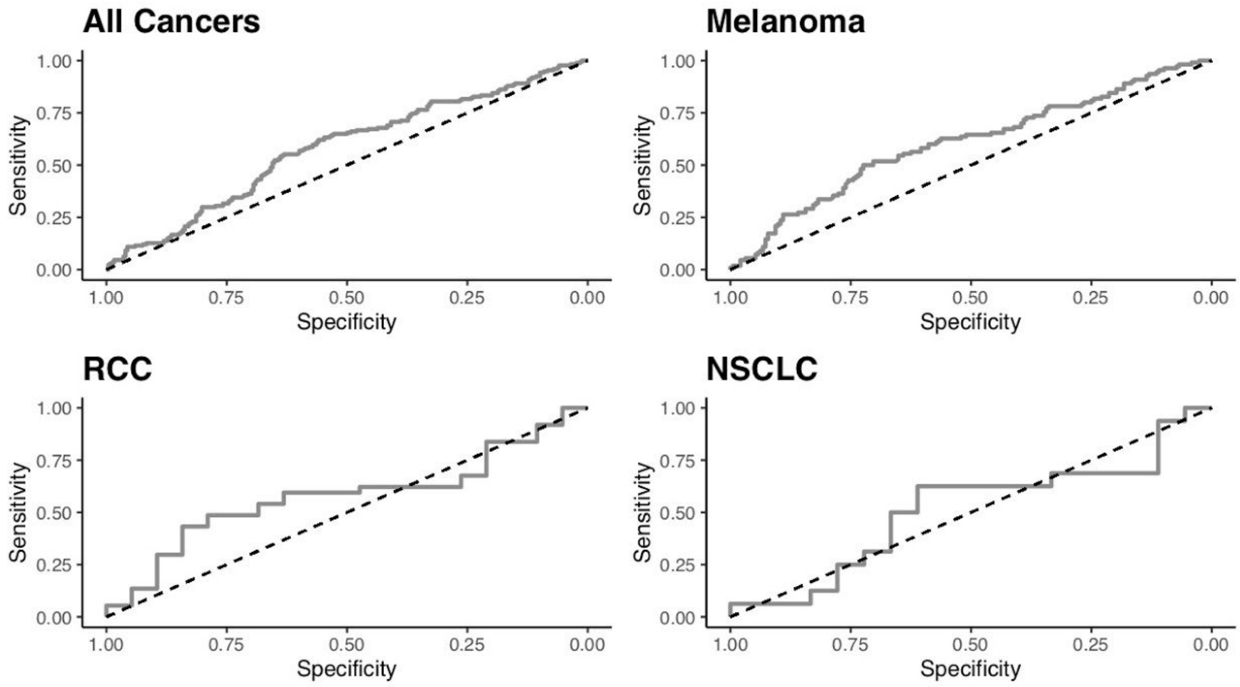

AUC by Coverage and Cancer Type

Cancer type	N	Coverage
All	431	0.581
Melanoma	302	0.606
RCC	57	0.578
NSCLC	34	0.510

Supplementary Figure S11: *Receiver operating characteristic curves of predictive capacity of Mbp of genomic coverage. The upper panels depict the true positive rate (sensitivity, y-axis) and false positive rate (1-specificity, x-axis) for genomic coverage across all probability thresholds. The four panels represent models for four different cohorts based on different subsets of patients: All Cancers, which includes all patients, and Melanoma, RCC, and NSCLC, which include only melanoma, RCC, and NSCLC patients, respectively. The table in the lower panel reports the area-under-the-curve (AUC) for coverage (right column) applied to a different cancer cohort (rows). RCC=renal cell carcinoma, NSCLC=non-small cell lung cancer.*

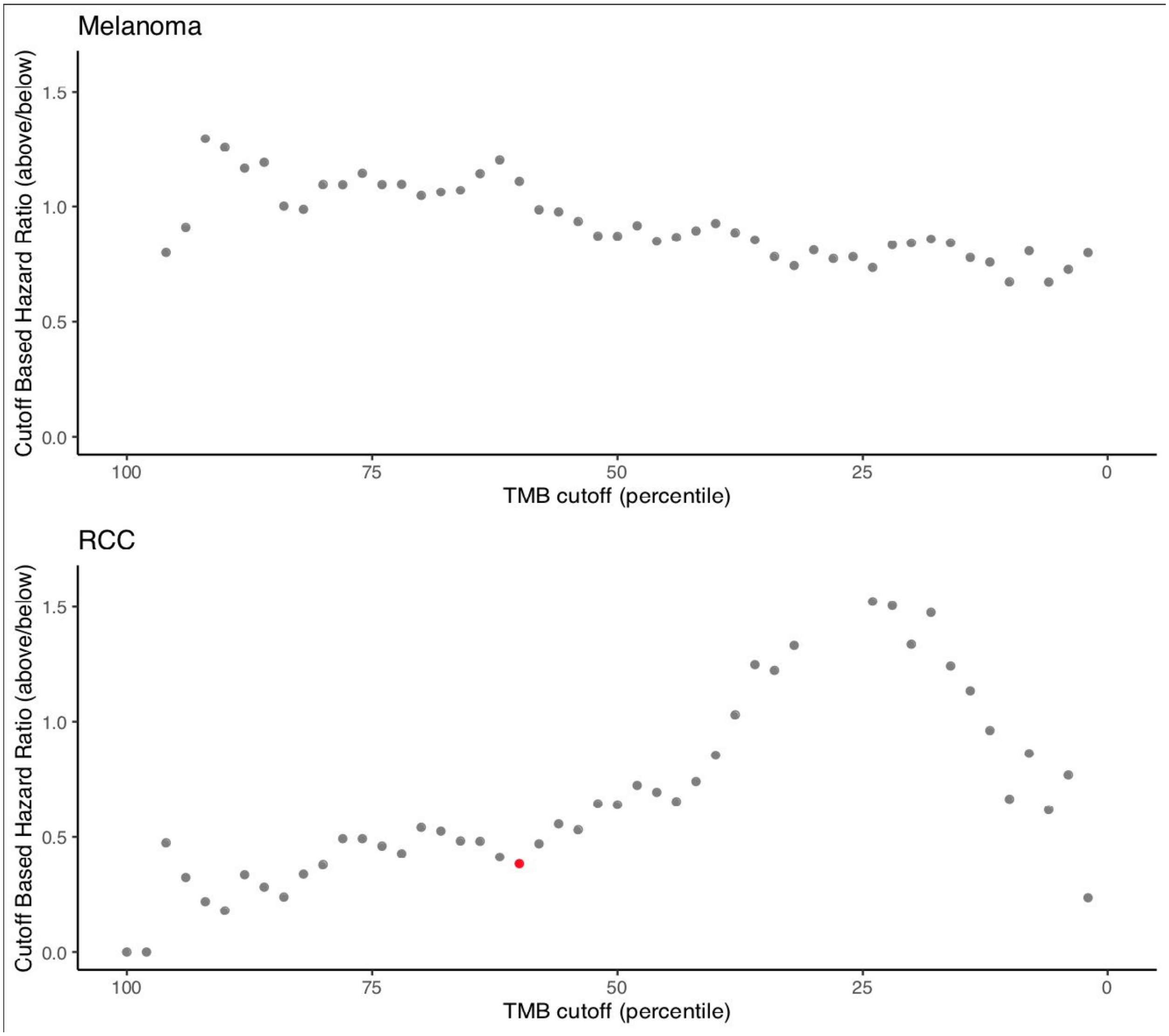

Supplementary Figure S12: *Variation in estimated hazard ratio based on TMB threshold selection. For melanoma and RCC separately, cox proportional hazard models were fit comparing patients above and below each TMB percentile cutoff at 2% intervals. The relative hazard ratio for those above the threshold compared to those below the threshold was plotted, with red representing models with corresponding unadjusted p-values < 0.05.*

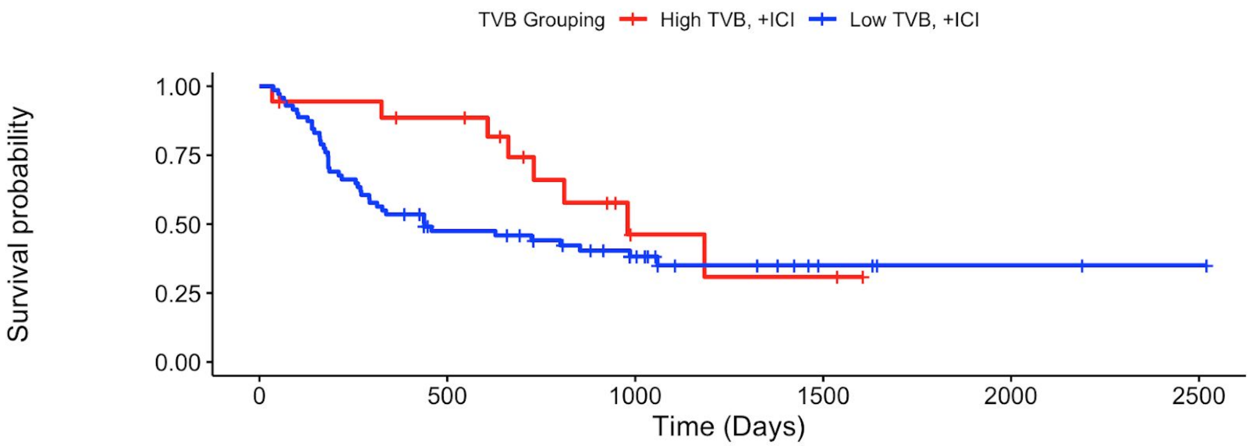

	0	500	1000	1500	2000	2500
High TVB, +ICI	18	14	3	2	0	0
Low TVB, +ICI	71	30	16	4	2	1

Supplementary Figure S13: *Overall survival among melanoma patients with high and low tumor variant burden (TVB). Kaplan-meier curves for the immunotherapy-treated patients with high TVB (≥80th percentile) and TVB burden (<80th percentile) are shown in red and blue, respectively. The underlying table corresponds to the number of patients at risk of death at each timepoint.*

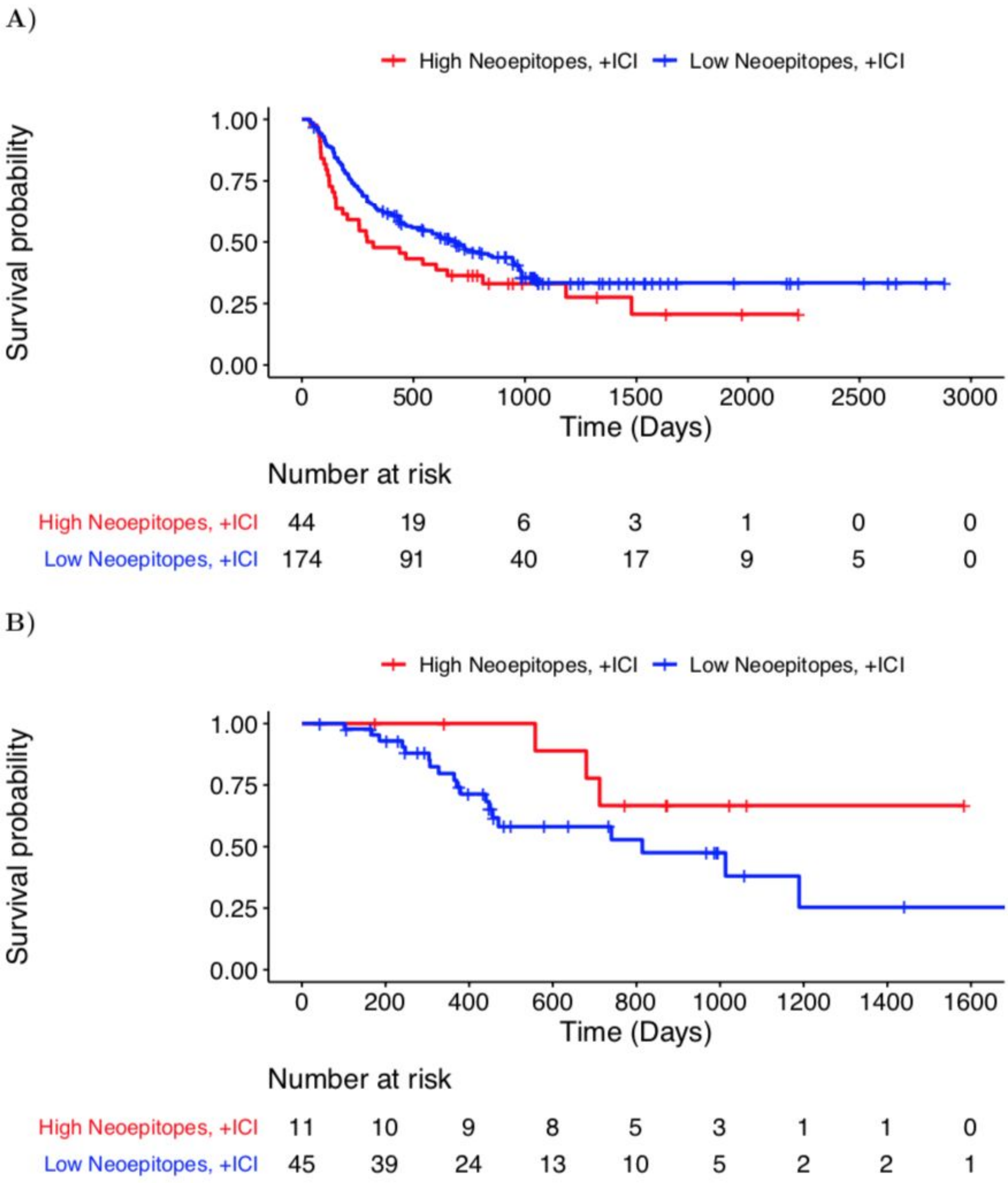

Supplementary Figure S14: *Overall survival among melanoma and renal cell carcinoma patients with high and low neoepitope burden. A) Overall survival among melanoma patients with high and low neoepitope burden. Kaplan-meier curves for the immunotherapy-treated patients with high neoepitope burden (≥80th percentile) and low neoepitope burden (<80th percentile) are shown in red and blue, respectively. The underlying table corresponds to the number of patients at risk for each timepoint. B) Overall survival among metastatic renal cell carcinoma patients with high and low neoepitope burdens. Kaplan-meier curves for the immunotherapy-treated patients with high neoepitope burden (≥80th percentile) and low neoepitope burden (<80th percentile) are shown in red and blue, respectively. The underlying table corresponds to the number of patients at risk for each timepoint.*

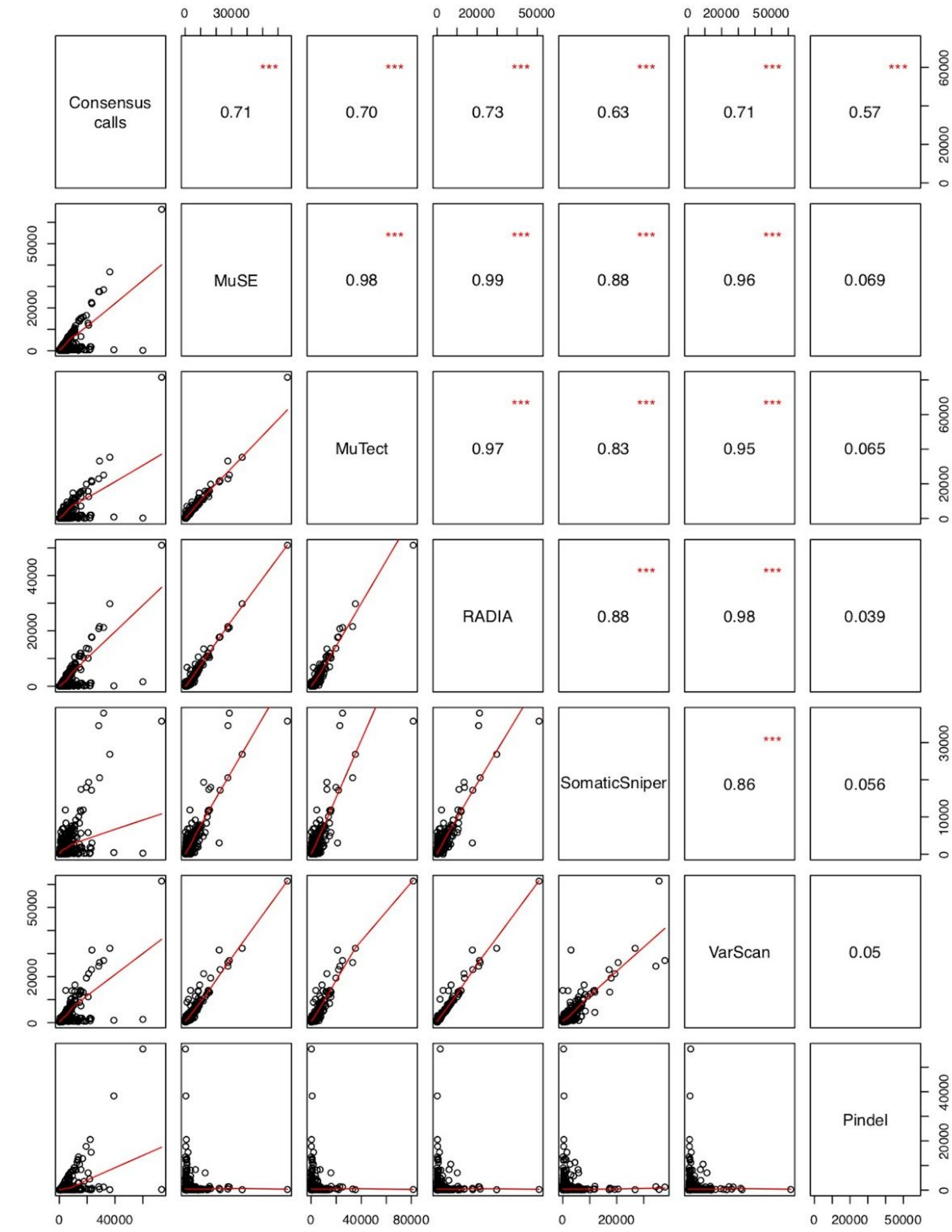

Supplementary Figure S15: *Pairwise differences in normalized total mutation burden as determined by 7 different computational approaches (see Methods). Each computational approach is identified along the diagonal panels, while the values in the upper panels denote the Pearson correlation coefficients between every pairwise combination of computational approaches (identified by corresponding row and column). The three red asterisks denote significant correlation at the p < 0.001 level. The scatterplots in the lower panels denote the TMB as calculated by each pairwise combination of computational approaches, with the x- and y-axes corresponding to the TMB calculated by the approach identified by the corresponding column and row, respectively; each open circle represents a single patient datapoint. Note that the red lines correspond to the best fit linear model.*

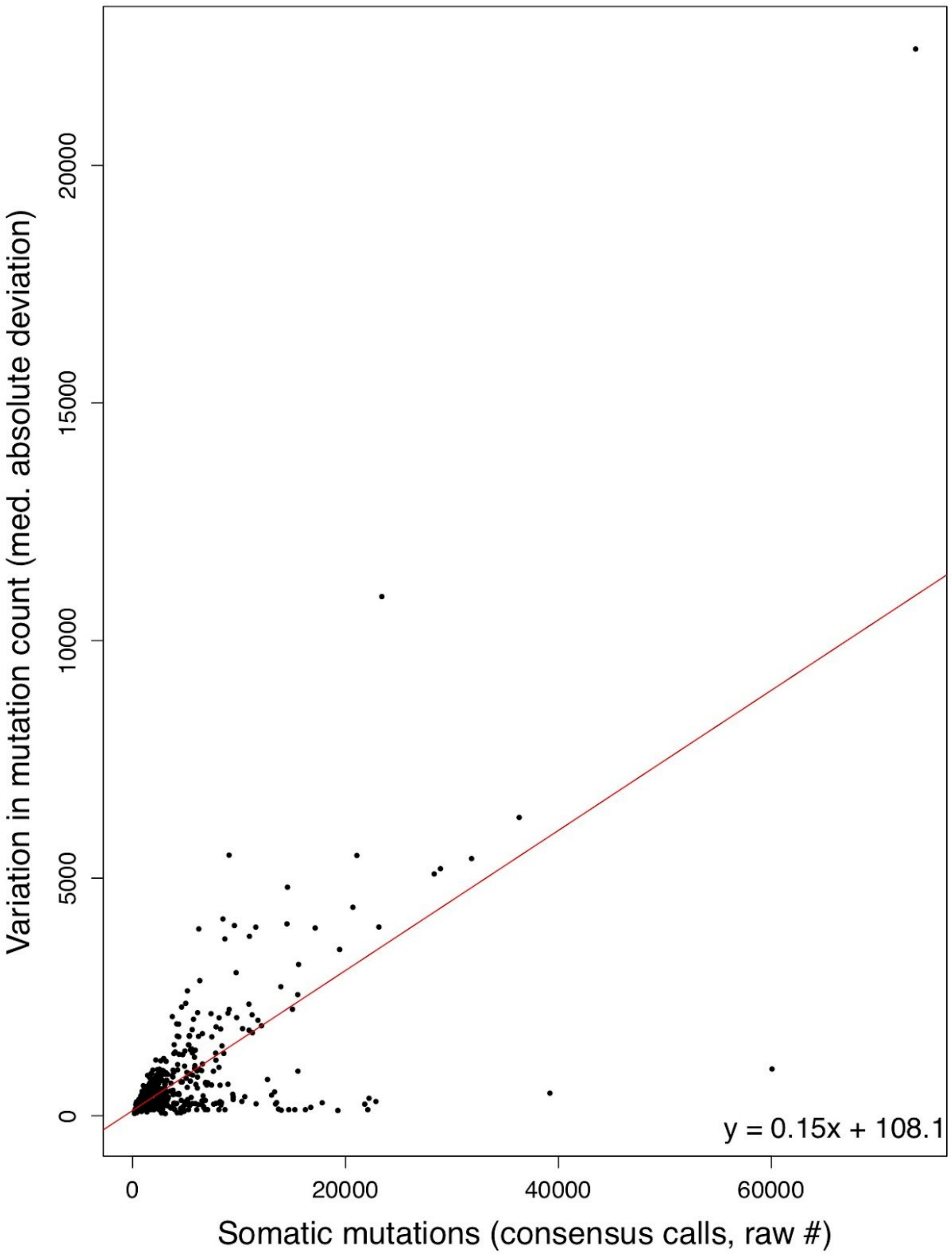

Supplementary Figure S16: *Variation in somatic mutation count increases with increased TMB from consensus variant calls. The median absolute deviation (MAD) in variant count across 6 variant calling tools used to determine consensus variant calls (y-axis, see Methods) increases with increasing TMB as determined by consensus calling (x-axis). The best fit line as determined by linear regression is shown in red, with its equation in the bottom right corner.*

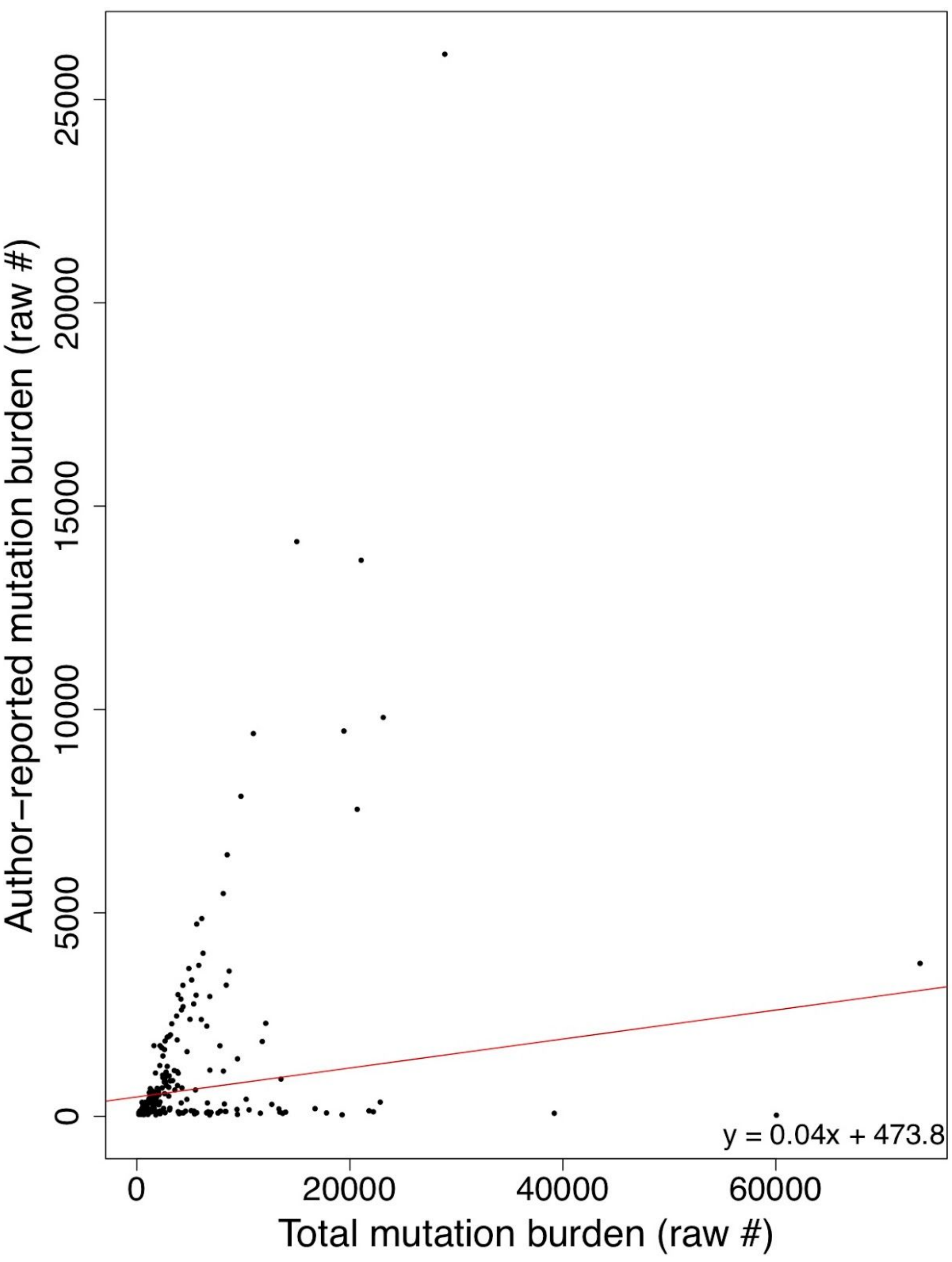

Supplementary Figure S17: *Author-reported total mutation burden correlates with consensus TMB. The total mutational burden as described by the authors of the original manuscripts from which the cohort derives (y-axis) correlates with our TMB derived from consensus variant calling (x-axis, Pearson product-moment correlation of 0.35, p = 1.99x10^{-7}). The best fit line as determined by linear regression is shown in red, with its equation in the bottom right corner.*

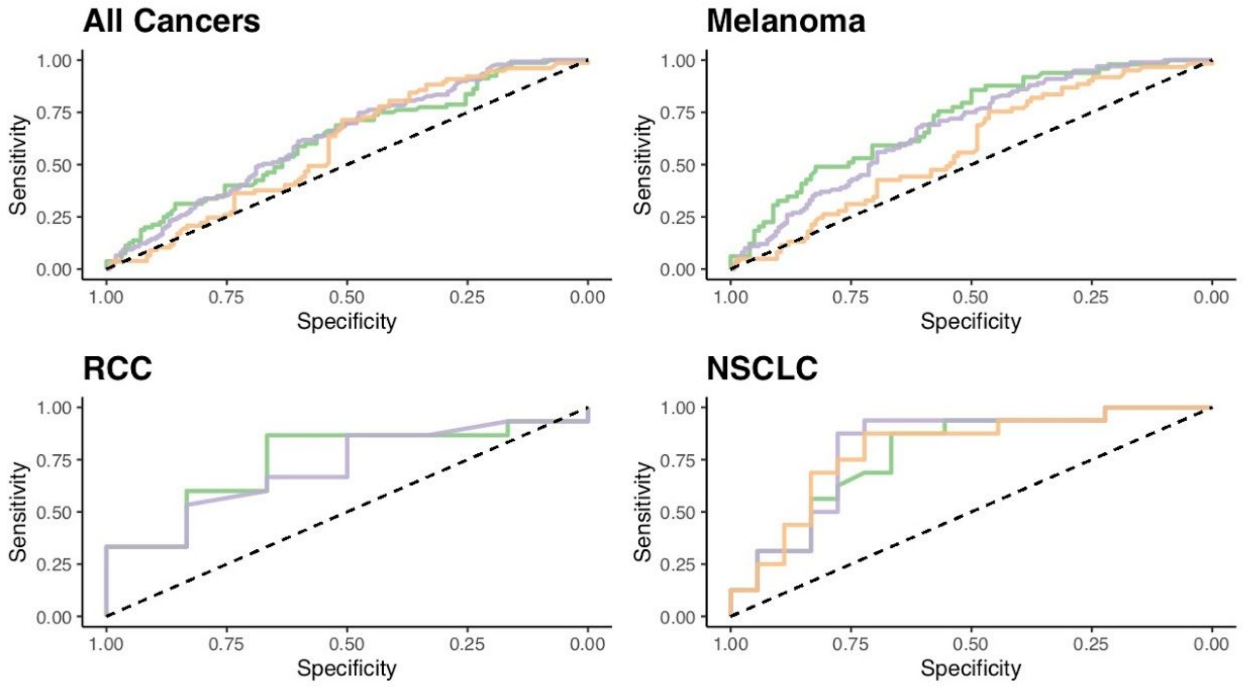

AUC by Author–reported Burden and Cancer Type

Cancer type	Total mutations	Nonsynonymous mutations	Neoantigens
All	0.620	**0.631**	0.587
Melanoma	**0.714**	0.677	0.582
RCC	**0.744**	0.711	NA
NSCLC	0.780	**0.809**	0.799

Supplementary Figure S18: *Receiver operating characteristic curves of predictive capacity of author-reported mutation and neoepitope burdens. The upper panels depict the true positive rate (sensitivity, y-axis) and false positive rate (1-specificity, x-axis) for each method across all probability thresholds. The four panels represent models for four different cohorts based on different subsets of patients: All Cancers, which includes all patients, and Melanoma, RCC, and NSCLC, which include only melanoma, RCC, and NSCLC patients, respectively. The table in the lower panel reports the area-under-the-curve (AUC) for each method (columns) applied to a different cancer cohort (rows), with colors above the methods indicating the color of the corresponding curve in the upper panels. Bold-faced values indicate the best value for each cancer cohort. RCC=renal cell carcinoma, NSCLC=non-small cell lung cancer.*

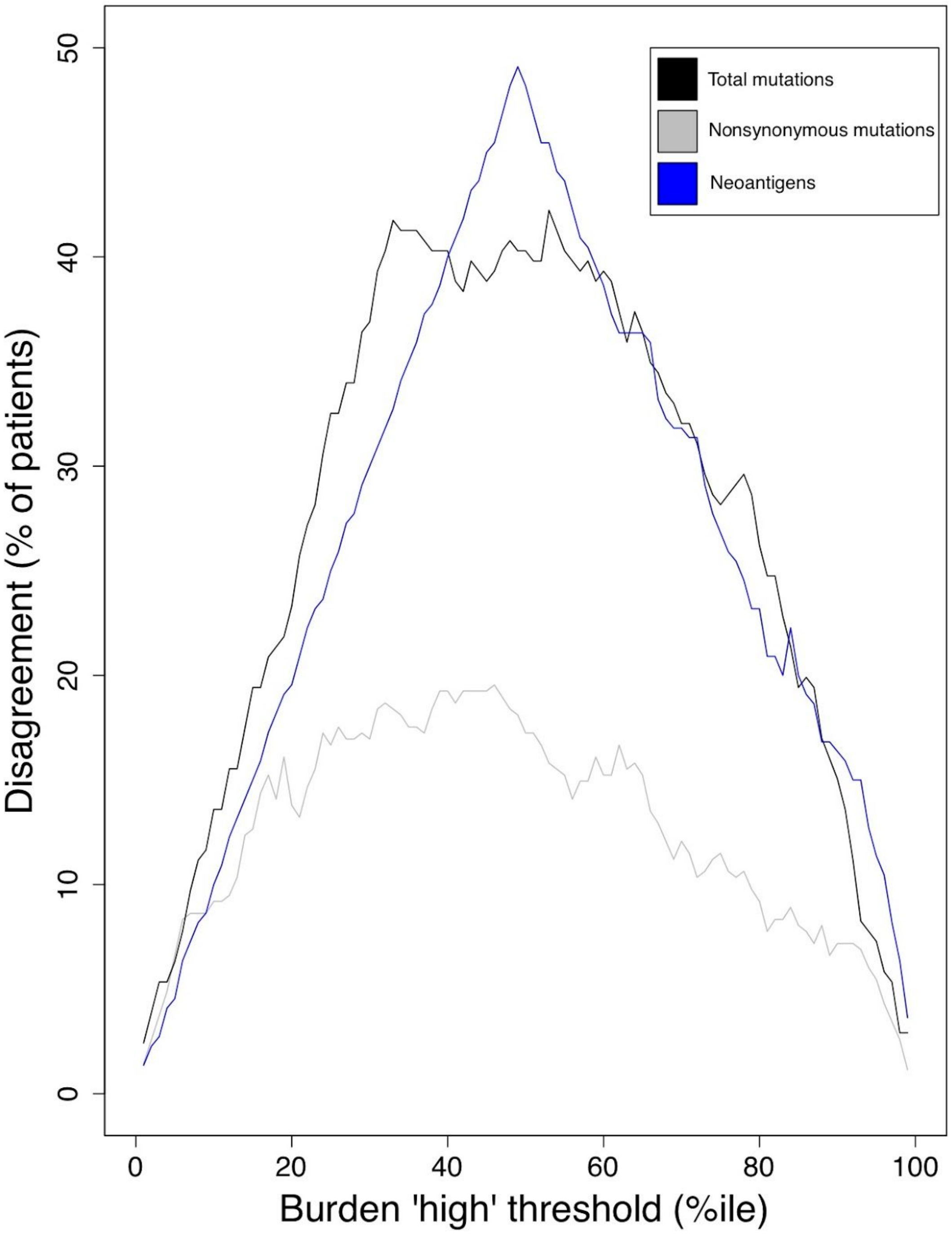

Supplementary Figure S19: *Cohort-level disagreement in classification of individual patients as TMB or neoepitope burden "high" v. "low". TMB and neoepitope burdens were calculated using a standardized consensus approach (see Methods) and were compared with author-reported values from the original cohort source studies. The overall disagreement between classifications of consensus and author-reported data (y-axis) was calculated using different percentile thresholds (x-axis) to classify each individual as e.g. TMB "high" or "low". This process was repeated for all mutations (black line), nonsynonymous mutations (gray line), and putative neoantigens (blue line).*

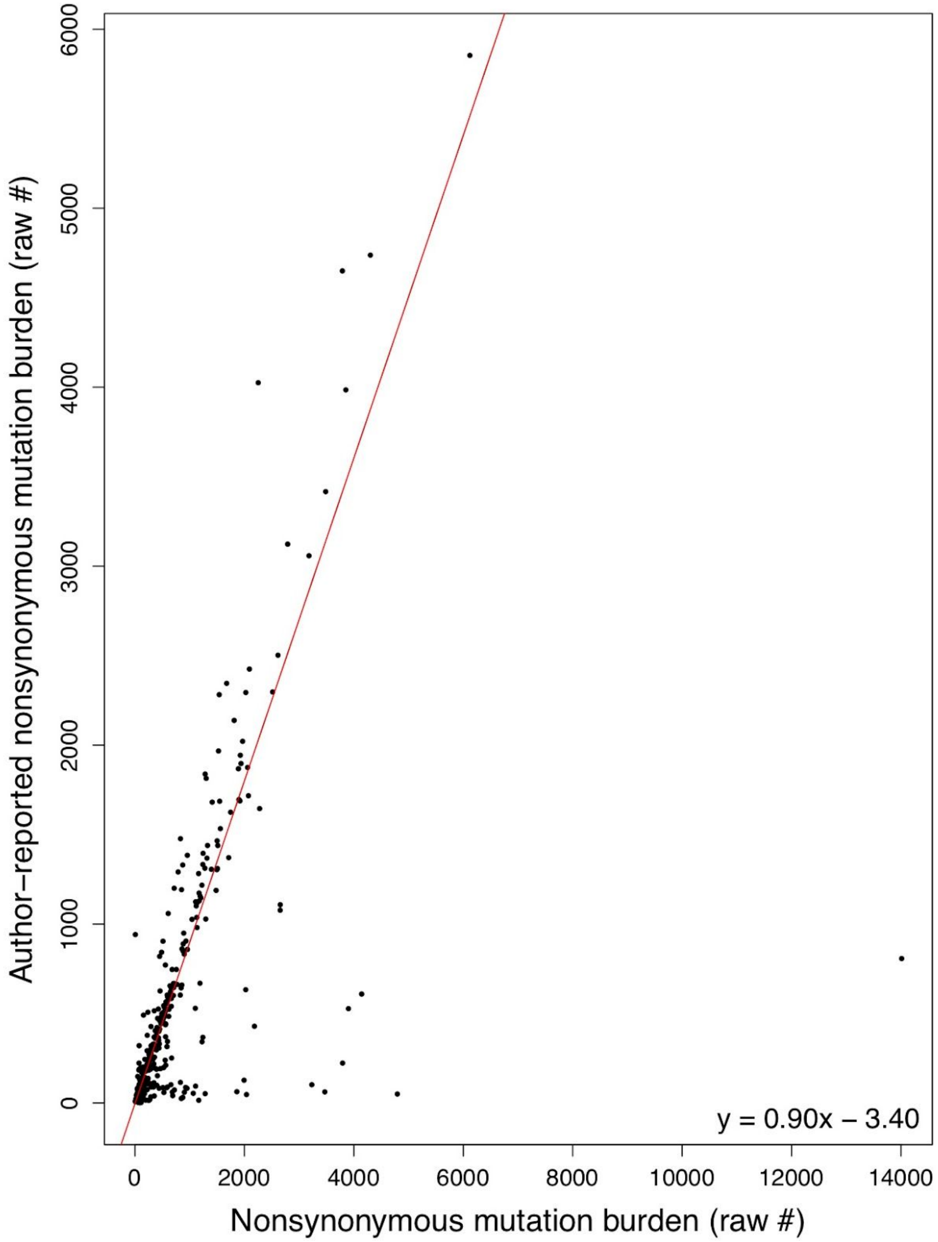

Supplementary Figure S20: *Author-reported nonsynonymous mutation burden correlates with nonsynoymous variants from consensus calling. The nonsynonymous mutational burden as described by the authors of the original manuscripts from which the cohort derives (y-axis) correlates with our consensus variant calling-derived nonsynonymous mutation burden (x-axis, Pearson product-moment correlation of 0.58, $p < 2.2x10^{-16}$). The best fit line as determined by linear regression is shown in red, with its equation in the bottom right corner.*

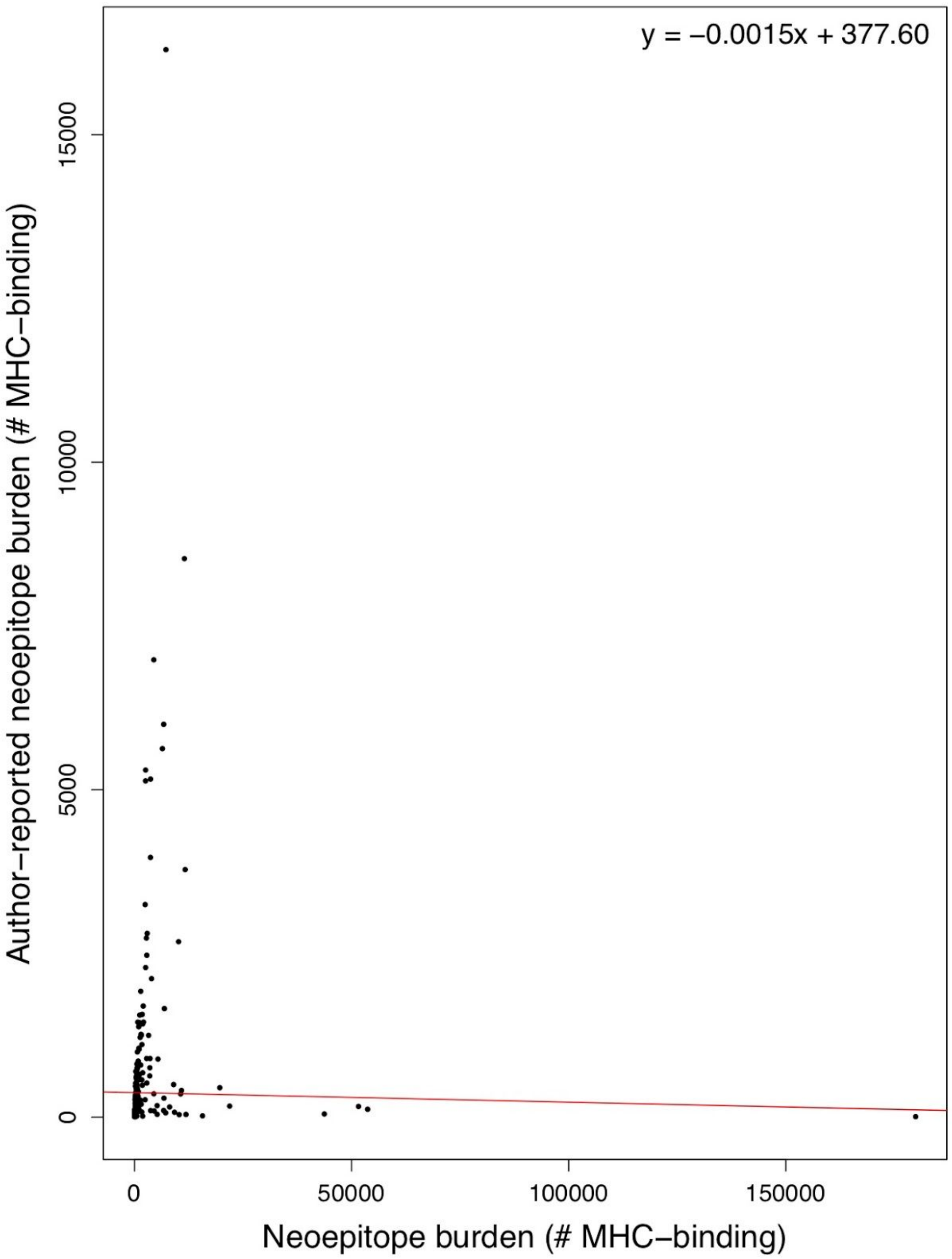

Supplementary Figure S21: *Author-reported neoepitope burden correlates with neoepitopes derived from variants from consensus calling. The neoepitope burden as described by the authors of the original manuscripts from which the cohort derives (y-axis) correlates with our consensus variant calling-derived neoepitope burden (x-axis, Pearson product-moment correlation of 0.026, p = 0.70). The best fit line as determined by linear regression is shown in red, with its equation in the bottom right corner.*

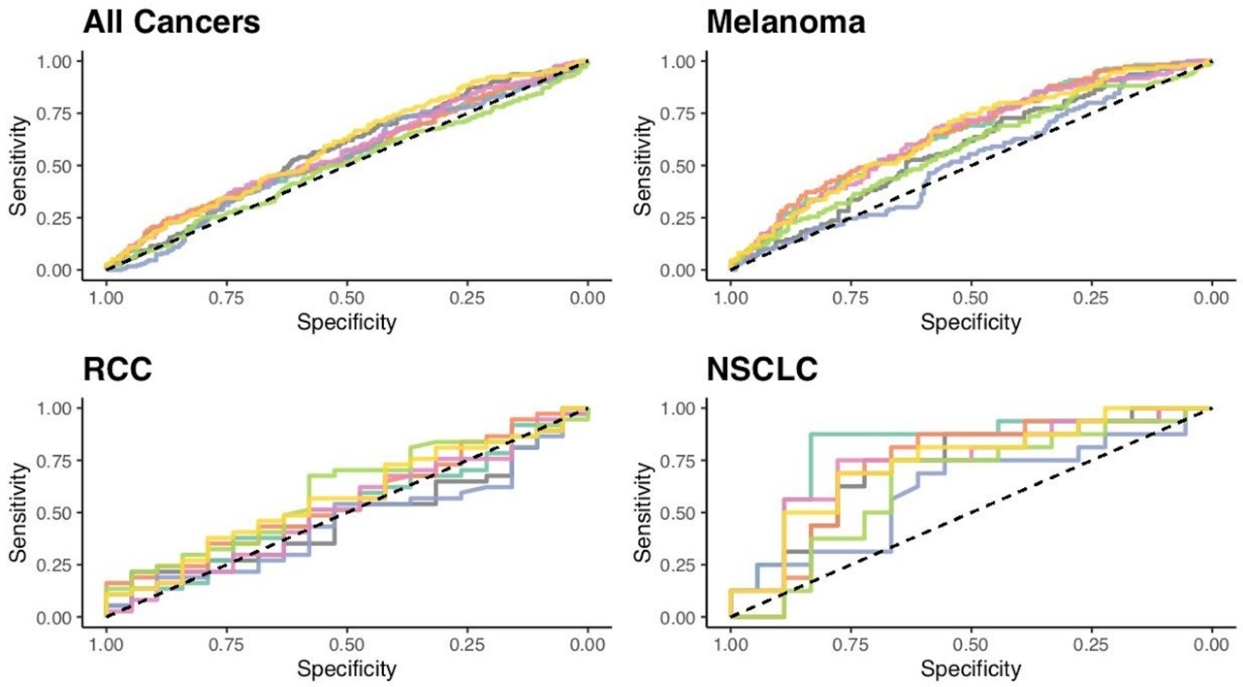

AUC by Mutation Burden and Cancer Type

Cancer type	N	Consensus	MuSE	MuTect	Pindel	RADIA	SomaticSniper	VarScan
All	431	0.572	0.556	0.555	0.533	0.568	0.499	**0.593**
Melanoma	302	0.582	0.655	**0.661**	0.524	0.647	0.581	0.649
RCC	57	0.477	0.522	0.561	0.454	0.515	**0.596**	0.569
NSCLC	34	0.726	**0.816**	0.726	0.613	0.743	0.642	0.740

Supplementary Figure S22: *Receiver operating characteristic curves of predictive capacity of TMB from 7 different variant calling methods. The upper panels depict the true positive rate (sensitivity, y-axis) and false positive rate (1-specificity, x-axis) for each method across all probability thresholds. The four panels represent models for four different cohorts based on different subsets of patients: All Cancers, which includes all patients, and Melanoma, RCC, and NSCLC, which include only melanoma, RCC, and NSCLC patients, respectively. The table in the lower panel reports the area-under-the-curve (AUC) for each method (columns) applied to a different cancer cohort (rows), with colors above the methods indicating the color of the corresponding curve in the upper panels. TMB as determined by consensus calling (see Methods) is compared to the individual variant calling tools used in consensus calling. Bold-faced values indicate the best value for each cancer cohort. RCC=renal cell carcinoma, NSCLC=non-small cell lung cancer.*

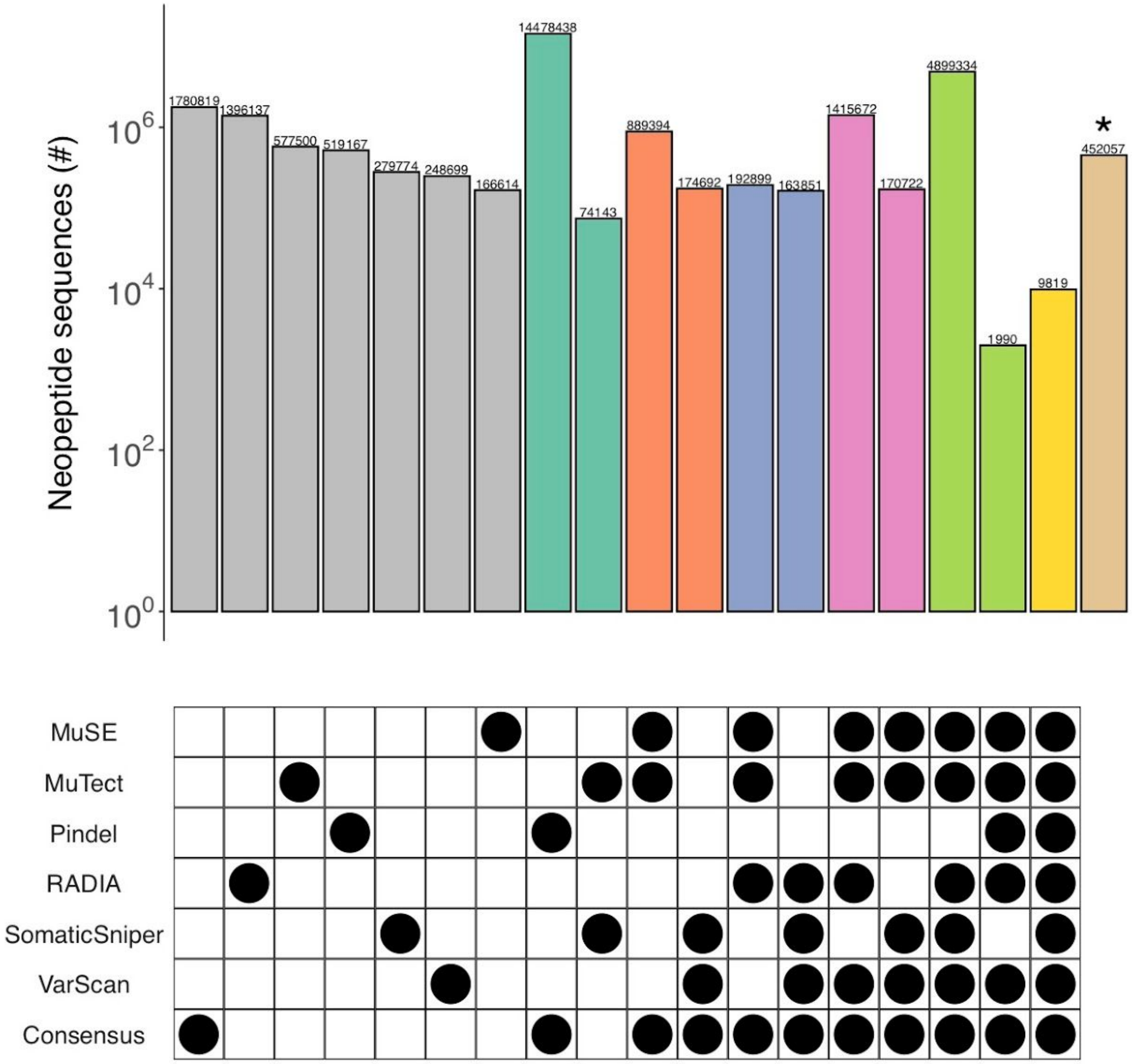

Supplementary Figure S23: *Detailed comparison of the complete set of neopeptide sequences predictions from MuSE, Mutect, Pindel, RADIA, SomaticSniper, VarScan, and consensus variant calling. Patterns of agreement or disagreement among groups of neopeptide sequences predicted from variants derived from different combinations of tools across all patients are shown along each column, and each row indicates the neopeptide predictions associated with variants from the indicated tool (e.g. the first column corresponds to neopeptides predicted only from Pindel variants). The number of neopeptides in each column (bar in upper pane) corresponds to the size of the subset predicted for variants from the indicated combination of tools (black circles in the bottom panel). Columns with gray bars represent neopeptides predicted from variants derived from only a single tool while columns with teal, orange, blue, pink, or green bars represent neopeptides predicted from variants derived from the most common two combinations of 2, 3, 4, 5, or 6 variant calling tools. The column with the yellow bar represents neopeptides predicted from variants deriving from all tools. The column with the brown bar (indicated by an asterisk) represents variants derived from less common combinations of 2-6 variant calling tools.*
